# Deep learning–assisted electron microscopy for apparent Z-height mapping of atomically dispersed catalysts

**DOI:** 10.1126/sciadv.aef7673

**Published:** 2026-11-13

**Authors:** Xiaocang Han, Yuan Meng, Qilong Cai, Yanhui Hong, Zanlin Qiu, Weiwei Hu, Shu Miao, Kangshu Li, Jun Zhao, Fanqi Meng, Xiao Hai, Zezhong Zhang, Jiong Lu, Jin Zhang, Xiaoxu Zhao

**Affiliations:** ^1^ School of Materials Science and Engineering, Peking University, Beijing 100871, China.; ^2^ Department of Chemistry, National University of Singapore, 117543, Singapore.; ^3^ DP Technology, Beijing 100080, China.; ^4^ JEOL (BEIJING) Co. Ltd., Beijing, China.; ^5^ AI for Science Institute, Beijing 100084, China.

## Abstract

Atomically dispersed catalysts (ADCs) offer maximal metal utilization and local atomic/electronic structures, yet resolving their actual atomic configurations using aberration-corrected scanning transmission electron microscopy (AC-STEM) remains fundamentally constrained by depth ambiguity and complex experimental noise. Existing STEM–machine learning (ML) approaches often rely on ideal simulations or manual annotations, limiting their transferability to realistic ADC images and causing ambiguity between isolated atoms, projected dimers, and small clusters. Here, we develop a physics-informed, domain-adaptive CycleGAN-UNet framework for depth-aware ADC analysis. CycleGAN translates height-labeled simulations into experimental-like images while preserving atomic coordinates and apparent Z-height labels, and a depth-supervised UNet performs simultaneous atom localization and discrete Z-height classification. Support-specific simulations further incorporate support-dependent background contrast, including uniform amorphous carbon (am-C), am-C with local thickness variation, and crystalline supports, thereby improving the applicability of the framework to different ADC imaging conditions. Across diverse metals (Mn, Fe, Cu, Pd, and Pt) and supports (am-C, black phosphorus, and polymeric carbon nitride), the framework achieves high atom detection precision (∼95%) and reliable apparent Z-height classification at ∼2-nanometer binning. By encoding depth-dependent beam broadening features, the framework converts conventional 2D projections into quantitative, apparent Z-height atomic maps. In geminal Pt/PCN catalysts, it resolves the loading-dependent transition from isolated Pt monomers to Pt_2_ sites and links these configurations to dehydrogenative aromatization activity. This framework transforms STEM images from projected atom maps into quantitative, depth-aware atomic descriptors for ADC structure–activity analysis.

## INTRODUCTION

Atomically dispersed catalysts (ADCs), featuring well-dispersed metal species such as isolated single atoms, dimers, and small assemblies, represent a major breakthrough in heterogeneous catalysis by maximizing metal atom utilization and exploiting their discrete electronic structures, which originate from the absence of ordered metal-metal bonds and the cationic/anionic character of the active sites (*1*–*3*). Despite their exceptional performance across thermocatalysis (*4*, *5*), electrocatalysis (*6*), and photocatalysis (*7*), achieving precisely designed atomic configurations and full metal atom utilization remains a formidable challenge (*8*). This difficulty largely stems from structural heterogeneity (*9*), characterized by the coexistence of single atoms, dimers, trimers, and more complex assemblies, as well as their atomic dispersion (*10*), encompassing interatomic distances (*11*, *12*), local coordination geometries (*13*, *14*), and embedded depths (*15*), all of which critically and coherently modulate catalytic behavior (*8*). For instance, the superior performance of Pt_2_ on mesoporous graphitic carbon nitride (mpg-C_3_N_4_), with more than 99% conversion in nitrobenzene hydrogenation, highlights how dual-atom configurations outperform single-atom catalysis (SAC) and nanoparticle counterparts by enabling efficient H_2_ dissociation and promoting product desorption (*16*). Therefore, a robust methodology that can resolve such subtle yet functionally decisive configurations is crucial not only for fundamental understanding but also for rational design of catalysts with superior performance (*17*).

With advancements in high-order aberration correctors, aberration-corrected scanning transmission electron microscopy (AC-STEM) has emerged as a critical technique complementary to ensemble spectroscopic methods (*18*), enabling direct spatial mapping of SACs at the atomic scale. Using annular dark-field STEM (ADF-STEM) imaging (*19*), where the intensity approximately scales with the atomic number to the power of 1.6 to 1.9, this technique allows clear categorization of atom dispersion states in ADCs, including monomers, dimers, clusters, as well as their spatial distribution features, proximity, and overall density with atomic-scale resolution (*16*, *20*). This, in turn, provides a structural basis for understanding and correlating catalytic performance (*21*). However, traditional histogram-based analysis of STEM images for ADC configurations is highly subjective and demands extensive, time-consuming manual intervention due to experimental variabilities—detector noises, scanning distortions, surface contamination, etc. (*22*). Consequently, accurate identification of single-atom, dimer, or complex sites in ADF images remains inefficient and error prone. Besides, because ADF images inherently provide limited resolution along the depth (*Z*) direction, they represent only two-dimensional (2D) projections of the three-dimensional (3D) ADC configurations. When signals from single atoms at different vertical positions are closely spaced, apparent “dimers” can arise from the projections of spatially separated atoms, making it difficult to distinguish true dimers from false ones. As a result, atomic configurations interpreted from ADF images may be underestimated or misidentified, particularly in complex systems containing closely spaced atoms or noisy backgrounds.

To establish a standardized and high-throughput framework for image informative analysis, computer vision and machine learning (ML), particularly deep learning, have been increasingly used in electron microscopy (*23*) for denoising (*24*), point defect detection (*25*, *26*) (vacancies, dopants, and substitutions), and crystal structure classification (*27*–*29*) (phases and stacking polytypes), achieving high accuracy and flexibility (*30*–*32*). In ADC microscopy, several atom identification approaches have also been developed (*33*, *34*). Convolutional neural network (CNN)-based models (*35*) have enabled automated single-atom detection in AC-STEM images, reducing operator-dependent bias and allowing large-scale statistical analysis of atom density, proximity, clustering, and dispersion. CNN-GMM-VAE statistical workflows (*36*) have further provided quantitative descriptions of metal-center organization, nearest-neighbor distances, multimers, surface coverage, and multimetallic sites. In parallel, synthetic data–trained segmentation models, such as AtomNet (*37*), reduce the dependence on manual annotation of experimental images and assist in quantifying projected monomer/dimer/trimer configurations in ADCs. Meanwhile, generative learning and image style transfer methods, such as pix2pix cGAN-based workflows in EMcopilot (*38*), have gradually emerged to bridge the simulation-to-experiment domain gap. These studies have substantially advanced high-throughput and standardized ADC image analysis.

Nevertheless, most existing ADC-specific methods remain primarily 2D: They detect projected atom coordinates and infer configurations from in-plane interatomic distances. Vertically separated atoms located at different depths within a porous, rippled, or thickness-varying support can therefore overlap in projection and be misinterpreted as in-plane “dimers” or “trimers” (*36*). This projection degeneracy is further complicated by support thickness variations, contamination, adsorbates, scan distortion, detector noise, and depth-dependent probe broadening, which perturb the expected ADF intensity scaling with atomic number (*39*, *40*). Recent studies on impurity-induced false positives, support-dependent contrast variations, and multidefocus imaging (*41*) further highlight that apparent bright spots and intensity variations cannot always be interpreted as straightforward chemical or configurational signatures. Therefore, a key unresolved challenge is to move beyond 2D atom counting and projected clustering statistics toward depth-aware ADC configuration analysis under realistic experimental imaging conditions. Generic synthetic data–trained segmentation models are effective for atom localization, but their 2D blob-like labels do not provide calibrated Z-height information. Physics-based simulations can supply height-labeled contrast features, yet ideal simulated images do not fully capture experimental support contrast, detector noise, scan distortion, contamination, and background heterogeneity. This motivates a framework that combines physics-based height-labeled simulations with experimental-domain adaptation.

Here, we address this gap by developing a physics-informed domain adaptation framework, CycleGAN-UNet. The simulated training data carry both in-plane atomic position labels and discrete apparent Z-height labels. CycleGAN transfers the textural statistics of experimental micrographs onto the simulated images while preserving the simulation-defined physical labels. The resulting pseudo-experimental data are used to train a depth-supervised UNet for simultaneous atom localization and apparent Z-height classification. This enables depth-aware ADC configuration analysis and helps discriminate the in-plane dimers from vertically separated monomers in focus-referenced STEM images. By integrating physics-informed height labels with experimental-domain adaptation, the framework achieves ∼95% precision of atom identification across a library of ADCs—including period four to six metals (Mn, Fe, Cu, Pd, and Pt) on diverse supports—under dynamic imaging conditions, and robustly distinguishes isolated monomers from vertically or laterally assembled configurations. In geminal Pt/PCN catalysts, the model directly differentiates Pt monomers from dimers (Pt_2_) and links these structural motifs with their markedly different activities in dehydrogenative aromatization. These results highlight the value of our framework as more than a characterization tool: It establishes a structure-performance bridge that enables predictive guidance for rational catalyst design.

## RESULTS

### Depth-dependent ADF-STEM contrast and projection ambiguity

To recognize ADC configurations efficiently and accurately, the availability of large-scale, high-quality, and well-labeled datasets—without relying on labor-intensive manual efforts (*29*)—is a crucial step for discerning subtle microstructural variations in atomic-resolution STEM images (*31*). To this end, the proposed framework uses a generative model—CycleGAN—to generate high-quality simulated STEM images paired with precise ground truth labels (sites and height) that closely mirror the intricacies of experimental images. A CNN based on the UNet architecture is trained using these synthetic images to accurately localize atomic sites and extract height information, enabling the accurate distinction of single atoms. Subsequently, by programmatically measuring interatomic distances through nearest-neighbor searches, the framework reliably identifies possible atomic configurations (Fig. 1A).

**Fig. 1.**
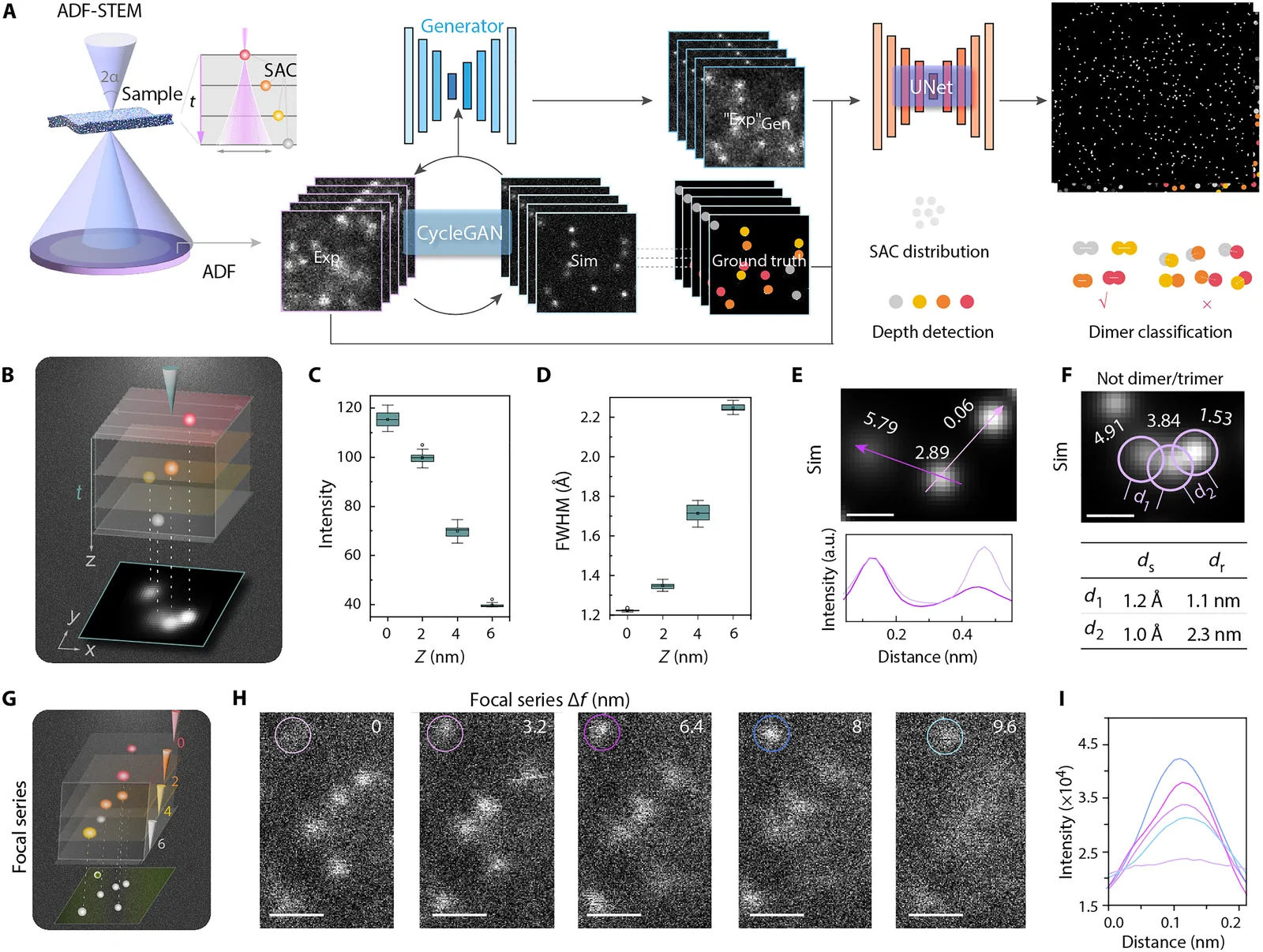
Single-atom site and height profiles in ADF-STEM images. (**A**) Schematic of the CycleGAN-UNet–based framework for single atom detection in ADF-STEM images. ADF-STEM imaging captures ADC at varying depths. Experimental (Exp) and simulated (Sim) images are bridged by a CycleGAN, generating pseudo-experimental data (“Exp”_Gen_) that align with simulated images annotated with ground truth atomic positions and depth. These annotated datasets are then used to train a UNet model for downstream prediction of ADC spatial distribution, depth classification, and dimer/trimer classification from experimental images. (**B** to **D**) Schematic illustration (B) and box plot demonstrating the intensity (C) and full width at half maximum (FWHM) (D) of Pt-SACs as a function of *Z* height. (**E** and **F**) Simulated STEM images of Pt atoms at varying heights without am-C and the corresponding intensity line profiles (E), illustrating intensity variations and the occurrence of false “Pt pairs” (F). The numbers in the images indicate atomic height information. The screen and real distances between Pt atoms are indicated by *d*_s_ and *d*_r_, respectively. (**G** and **H**) Schematic illustration (G) and through-focal series ADF-STEM imaging (H) at defocus values of 0, 3.2, 6.4, 8, and 9.6 nm. (**I**) Corresponding intensity line profiles from the Pt atom in (H) marked by circles. Scale bars, 0.2 nm [(E) and (F)] and 0.5 nm (H).

Although commonly noted, the impact of focus value on atomic visibility is often underestimated in STEM imaging of ADCs (*42*). Specifically, the intensity of single atoms decreases markedly with increasing distance from the focal plane due to variations in support thickness or differences in embedding depth, especially for porous or rippling supports (*43*, *44*). This pronounced attenuation along the optical (depth) axis primarily arises from the limited depth of focus and the reduced electron probe-atom interaction as defocus increases (*45*). To quantitatively characterize the depth-dependent attenuation of atomic contrast, we performed a statistical analysis of the intensities of representative Pt-SACs located at various depths in simulated images, with the electron beam focus fixed at the top surface (Fig. 1B). A clear inverse relationship between intensity and beam broadening as a function of *Z* height was observed (Fig. 1, C and D), consistent with beam divergence effects during propagation along the optical axis (*40*). Atoms located deeper (e.g., at 6 nm) beneath the focal surface exhibit broader full width at half maximum (FWHM) with markedly reduced intensity. To specifically assess focus-dependent beam divergence, the simulated model excluded the uniform light-elemental support for heavy Pt atoms, as ADF contrast variations from such a support are expected to be weaker than the defocus-induced changes in Pt atomic profiles (*41*). Simulated STEM images and corresponding intensity line profiles illustrate the contrast variations along the *Z* height (Fig. 1E). Notably, atoms in close lateral proximity within the imaging plane—commonly referred to as dimers and trimers—are, in reality, isolated single atoms separated by the substrate supports (Fig. 1F), using the 2.8-Å Pt-Pt bonding length in crystalline Pt as a threshold (*37*). Even for geminal metal pairs on the polymeric carbon nitride (PCN) substrate, dimers located at different heights can still confound configuration identification when the atoms are closely aligned in projection (*12*, *16*). Thus, precise depth localization becomes crucial to discriminate genuine isolated atoms from fake atomic assemblies that are merely aligned along the beam direction. In the ideal simulated optical system, the intensity of atoms located 6 nm away from the focal plane is approximately one-third of that of atoms at the focal plane—barely distinguishable in STEM images, even when the effects of the substrate and instrumental noise are excluded.

The depth-dependent intensity variation of Pt-SACs on an amorphous carbon (am-C) substrate was demonstrated using through-focal series (*45*) STEM imaging (Fig. 1G). As the defocus changes, individual Pt atoms remain visible across adjacent frames, but their peak intensities, normalized to the maximum value, vary markedly from 0.27 to 1.0 (Fig. 1, H and I). Specifically, in the frames taken at focus values of 0 and 3.2 nm, Pt atoms located at the center of the image remain in focus. In the next two snapshots (focus values of 6.4 and 8 nm), they become increasingly underfocus (denoted by positive values). On the other hand, the Pt atom located in the top-left corner of the image comes into focus when the focus value is set between 6.4 and 8 nm, with marked changes in intensity and a decrease in FWHM from 0.34 to 0.12 nm (Fig. 1I), suggesting that this Pt atom is likely anchored near the bottom surface of the am-C substrate (Fig. 1G). Under our imaging conditions, both experimental and simulated results support an apparent Z-height classification interval of ∼2 nm (Fig. 1H and fig. S1). At defocus values exceeding 6.5 nm, the contrast of individual atoms degrades markedly, rendering them nearly invisible (fig. S1) (*46*). The true defocus value was calibrated experimentally (fig. S2). Beam spreading increases as the focal plane moves away from the sample, both under underfocus and overfocus conditions (fig. S3). Therefore, the key conclusions can be drawn: (i) Identification of SACs at large underfocus/overfocus values (>8 nm or <−8 nm) remains challenging due to substantial intensity modulation and delocalization effects; (ii) intensity and FWHM are valid quantitative descriptors for inferring the apparent height of individual SACs.

However, in practical catalysts and imaging systems, noise and substrates may contribute to intensity variations, as demonstrated by the broad overlaps in the FWHM of the simulated dataset—with noise and uniform am-C substrate—across different height levels (fig. S4). Collectively, these factors further underscore the challenges associated with accurately detecting ADCs in experimental scenarios, where the presence of noise can exert a pronounced influence on the detection outcomes.

### CycleGAN-UNet workflow for data generation and atom detection

To achieve high precision in SAC site and height identifications, a learning model (CycleGAN) (*47*) was used to generate high-quality simulated STEM datasets that closely resemble the style of experimental STEM images (Fig. 2A) while precisely preserving their atomic positions. During the generation process, both experimental and simulated data were segmented to expand the training datasets for the subsequent atom identification model, UNet-based CNN. Given the distinguishable contrast differences across depth ranges in ADF-STEM images (Fig. 1, C and D, and figs. S1 and S4), a height step of 2 nm and a total depth range of 6.55 nm were selected.

**Fig. 2.**
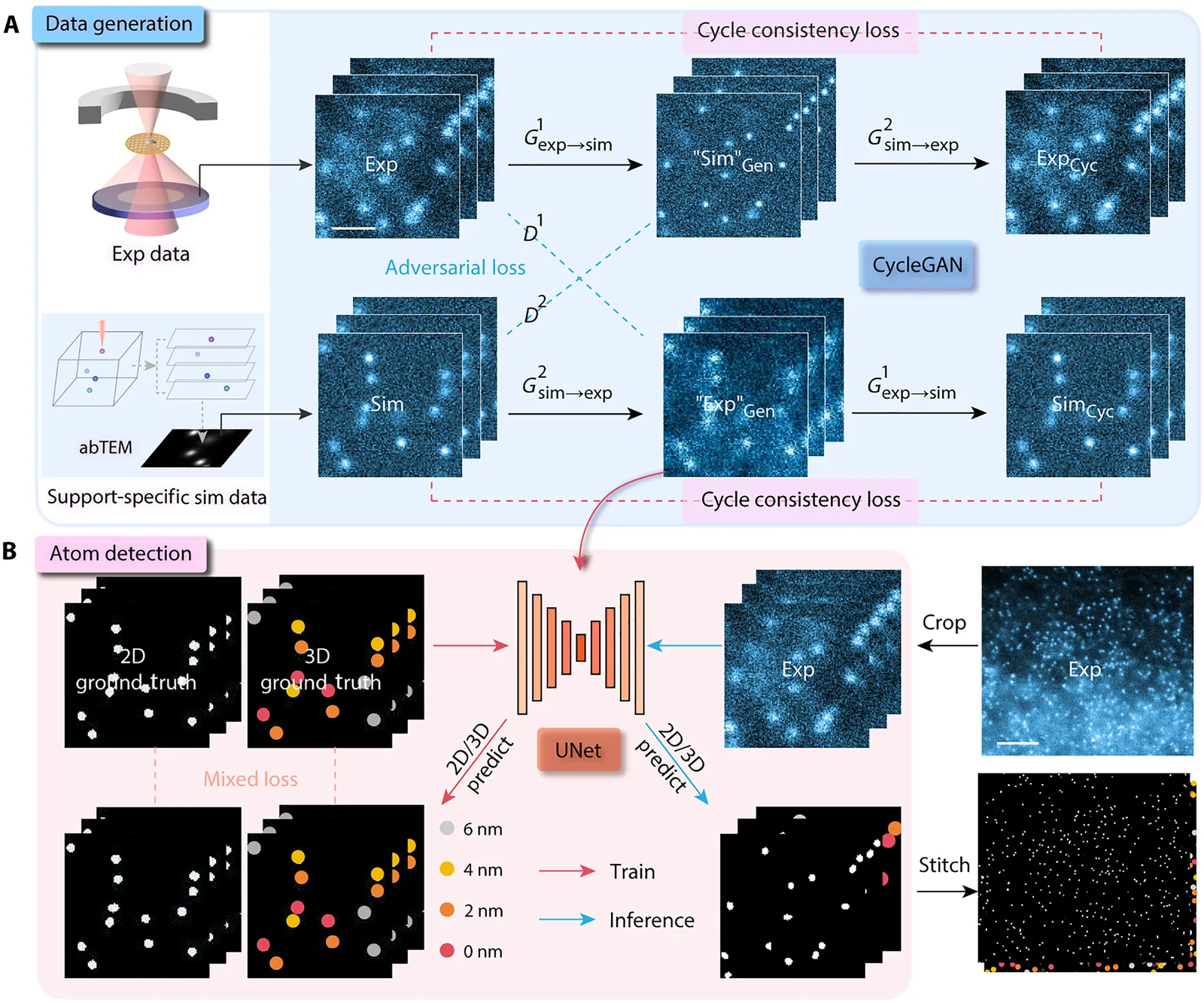
Workflow of data generation and atom detection via GAN-UNet. (**A**) Data generation and domain translation using CycleGAN. Experimental (Exp) and simulated (Sim) STEM images are used to train a CycleGAN model that learns bidirectional mappings between the two domains. The CycleGAN comprises two generators ($G_{\text{exp}\to \text{sim}}^{1}$ and $G_{\text{sim}\to \text{exp}}^{2}$) and two discriminators ($D_{\text{exp}}^{1}$ and $D_{\text{sim}}^{2}$) which are optimized through adversarial and cycle consistency losses, enabling the simulated data to acquire experimental-like image characteristics. (**B**) Atom detection using UNet. The CycleGAN-translated images are used to train a UNet model for predicting atomic distributions (red arrows). The network is supervised using 2D/3D ground truth data, optimizing a mixed loss function to accurately predict atomic coordinates in both 2D/3D spaces, with the 3D predictions capturing different depths. During inference, experimental images are cropped, processed, and stitched to reconstruct the atomic distribution map (blue arrows). Scale bars, 0.5 nm (A) and 2 nm (B).

The precise identification of the position and height of SACs based on the CycleGAN and UNet (denoted as GAN-UNet) model is featured by data generation (Fig. 2A) and atom detection (Fig. 2B), which was divided into four steps: (i) training CycleGAN with segmented simulated and experimental STEM images; (ii) generating realistic simulated datasets learning from experimental datasets; (iii) training UNet with realistic simulated images; (iv) auto-identifying SAC sites and heights in experimental STEM images and stitching. For apparent *Z*-height identification, support-specific simulated datasets are required so that the model can learn depth-dependent atomic features under support-matched background contrast. Dimers and trimers were identified using adjustable thresholds for interatomic spacing.

### CycleGAN domain translation and robust atom localization

Unsupervised CycleGAN (*25*), benefiting from its cycle consistency constraint and bidirectional independent generator design, allows simulated images to acquire experimental-like noise, background texture, and detector-related contrast (Fig. 3, A and B). The performance of the CycleGAN-generated image (denoted as GAN) quality is evaluated via grayscale distributions of different simulated images, including the noise-free simulation (sim), the manually noise-added (Poisson and Gaussian) simulation (denoted as manu), and an experimental image (exp) (Fig. 3A). Metrics such as Kullback-Leibler divergence (*D*_KL_) and Fréchet inception distance (FID) were introduced to quantitatively measure the similarity between experimental and three simulated images (Fig. 3B). *D*_KL_ values for grayscale distributions were 2.72, 1.16, and 0.19 for the sim, manu, and GAN, respectively, suggesting the highest similarity between the CycleGAN-generated image and experimental data (the lower the value, the greater the similarity). This is further supported by a lower FID score (FID = 46), demonstrating the success of the CycleGAN model in image style/feature learning. Although unsupervised image-to-image translation (*48*, *49*) also achieves high image similarity (FID = 41, *D*_KL_ = 0.08), its shared latent space assumption may limit its generalization in specific scenarios (fig. S5). In contrast, the architecture of CycleGAN enhances its robustness in tasks involving unpaired data and pronounced domain discrepancies, enabling more effective cross-domain translation without the need for paired training examples (*47*).

**Fig. 3.**
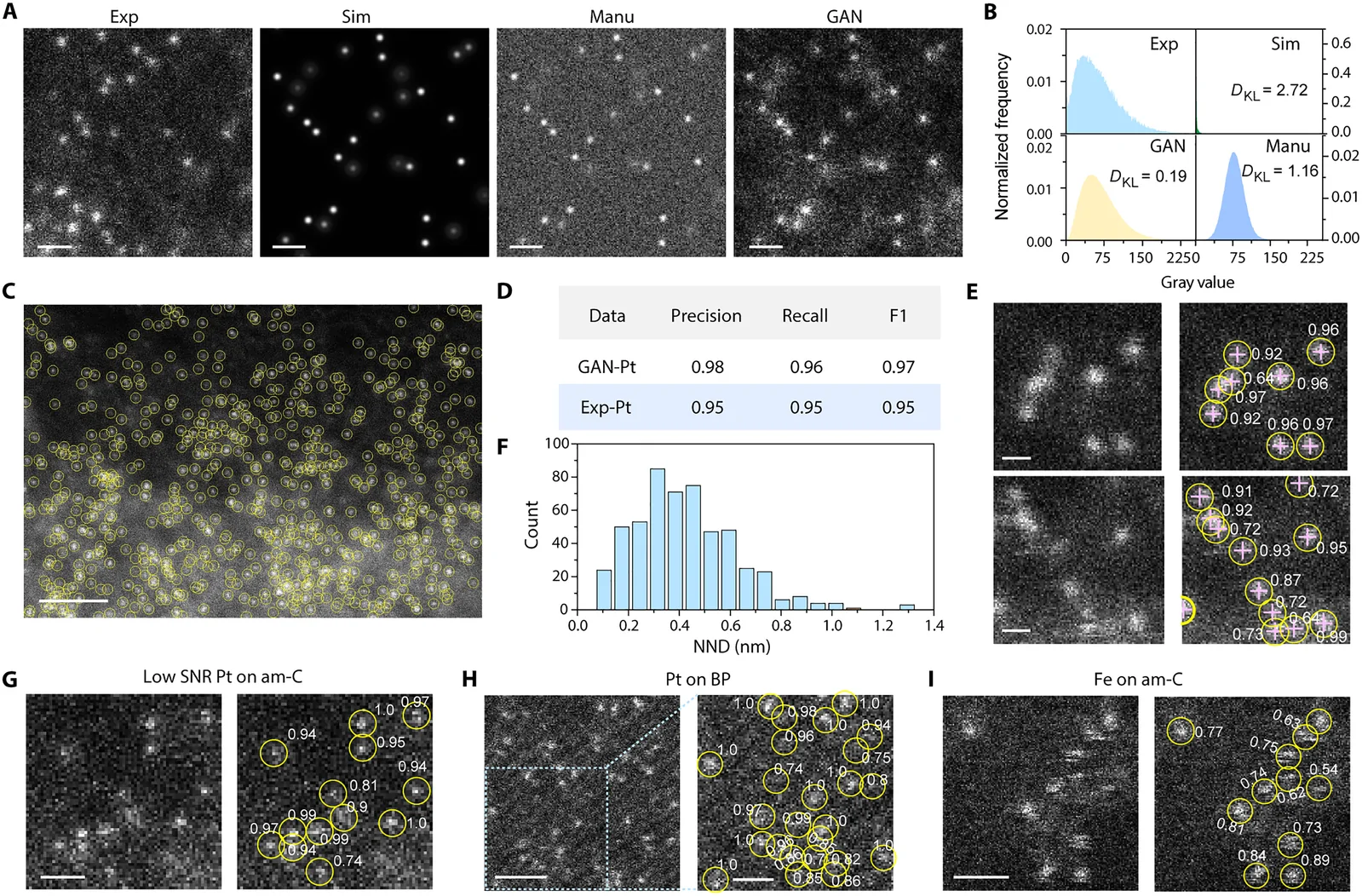
Robust performances of the GAN-UNet model for automatic SAC detection. (**A**) Experimental (Exp), noise-free simulated (Sim), manually noise-added simulated (Manu), and CycleGAN-generated (GAN) images of Pt single-atom catalysts. (**B**) Grayscale distribution of Exp, Sim, Manu, and GAN images, with Kullback-Leibler divergence (*D*_KL_) values quantifying the similarity among the image datasets. (**C**) Automatic detection of Pt single atoms in experimental STEM images containing highly dispersed Pt-SACs using GAN-UNet–based models. (**D**) Quantitative evaluation (precision, recall, and F1 scores) of the detection algorithm for GAN-Pt and Exp-Pt datasets. (**E**) GAN-UNet identification on Pt-SACs and overlapped Pt atomic clusters, well consistent with manual annotations (marked by pink crosses). (**F**) Calculated nearest-neighbor distance (NND) distribution of detected atoms from (C). (**G** to **I**) Performances on a variety of SAC samples verifying the reliability and generality of the GAN-UNet model, including low–signal-to-noise ratio (SNR) Pt on am-C (G), Pt on BP (H), and Fe on carbon supports (I). Yellow circles indicate the detected single atoms, with the corresponding confidence levels annotated on the images. The input STEM image in (H) was adapted from supplementary figure S11b of Chen *et al*. (*56*), with permission from Wiley. Copyright 2021 Wiley-VCH GmbH. The image was cropped and contrast-adjusted for visualization. Scale bars, 0.5 nm [(A), (G), (I), and right of (H)], 2 nm (C), 0.2 nm (E), and 1 nm [left of (H)].

The experimental image of Pt-SACs on am-C was used as a test case to evaluate the performance of CycleGAN-processed high-quality simulated images as training datasets for the atom detection UNet model (Fig. 3C and table S1). The results demonstrated that large-scale, high-density Pt-SACs were widely identified on the basis of expert judgment (Fig. 3C and fig. S6). Metrics such as precision, recall, and F1 scores were used to assess the model performance against human annotations (Fig. 3D). Impressively, this framework achieved approximately 95% or higher precision and recall in identifying Pt atoms across both experimental and simulated datasets, demonstrating consistent detection performance for Pt-SACs on substrates with varying contrast and homogeneity. Magnified areas in the identification results display the confidence level of each detected site, providing the solid foundation for SAC identification (Fig. 3E), highlighting the robustness of SAC detection, even for small interatomic distances (*d*_s_ ∼ 0.8 Å) and under conditions of severe scan distortion (marked by arrow). The nearest-neighbor distance (NND) distribution reveals that most of the single atoms are densely distributed on the catalyst substrate (Fig. 3F). A discrete pattern with a higher concentration of atoms at smaller distances suggests that atoms are placed in a more local, nonrandom arrangement, which is critical for the catalytic activity of SACs (*10*). The estimated density of 4.03/nm^2^ remained unchanged across multiple detections by this model, confirming its robustness. Further validation of the GAN-UNet model was performed using experimental datasets with low signal-to-noise ratio (SNR) images, including Pt-SACs on am-C (Fig. 3G), Pt-SACs on crystalline black phosphorus (BP) (Fig. 3H), Fe-SACs on carbon supports (Fig. 3I), as well as Pd and Mn SACs (figs. S7 to S12). The model verifies high detection precision across diverse systems, even for elements with atomic numbers as low as 25 and atomic site densities as high as 14 atom nm^−2^. Furthermore, it excels in identifying SACs under challenging conditions, such as low SNR or severe scan distortions.

It is noteworthy that the accuracy of the inference model’s identification depends on the pixel size used to generate realistic STEM simulated images. To enhance the universality of the UNet models, the generation of CycleGAN-processed simulated images was performed using pixel sizes ranging from 0.1 to 0.3 Å. In addition, the density of SACs in the training datasets plays a crucial role in the atom detection model, so we set the atomic density ranging from 0.4 to 1.6 atoms/nm^3^ in the training datasets with random height distribution (fig. S13). The recognition results demonstrated the exceptional accuracy, robustness, and generalizability of the GAN-UNet model across varying SNR levels, substrates, densities, and atomic numbers. However, the true NND can only be obtained by considering the height information. Atoms appearing closer than 2.8 Å in projection may, in fact, not be dimers (Fig. 1F).

### Support-specific apparent *Z*-height classification and uncertainty analysis

Furthermore, we extend the framework from mere site localization to *Z*-axis classification. By training the network to recognize specific intensity attenuation and beam-broadening signatures [Airy-like variations (*50*)] associated with defocus, the model can reliably resolve the height levels of ADCs in STEM images—an essential capability for distinguishing true dimers from vertically separated monomers and for establishing structure-property correlations (*9*). For height classification, the GAN-UNet model was trained on simulated datasets constructed at discrete depths (e.g., 0, 2, 4, and 6 nm; Fig. 4A). The predicted results on CycleGAN-translated images confirm accurate height assignment across these levels (fig. S14). The model distinguishes single atoms from vertically stacked atomic pairs with very small projected separations (*d*_s_ < 2.8 Å)—that human experts routinely misclassify as dimers (Fig. 4A). This depth awareness effectively eliminates human-induced inconsistencies and substantially improves the reliability of ADC configuration identification. To verify that CycleGAN does not distort the depth-dependent atomic profiles, we compared the intensity-FWHM distributions of simulated Pt atomic blobs before and after CycleGAN processing (fig. S15). The preserved depth-dependent trend suggests that domain translation mainly enables the simulated images to acquire experimental-like texture and background statistics, while the Z-height labels and physical features used for apparent Z-height classification are retained from the original simulations.

**Fig. 4.**
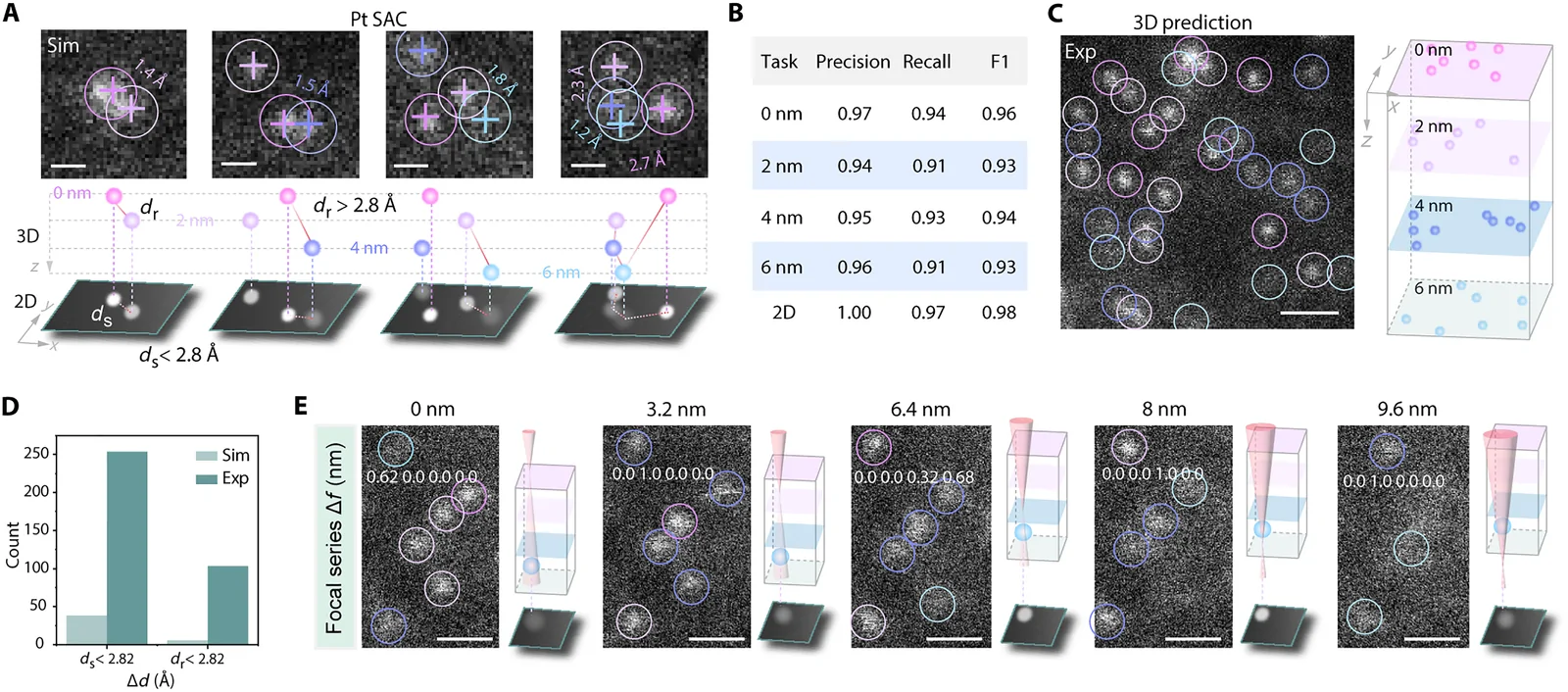
Generalizability of the GAN-UNet model for single-atom height classification. (**A**) Identification of the “dimer” formed by two vertically separated single atoms with different heights in simulated STEM images. Colored circles are model predictions, closely resembling the ground truth marked by color crosses. The bottom panel illustrates the 3D distributions of Pt-SACs and the relation between the 2D projected separation *d*_s_ and the true 3D distance *d*_r_. (**B**) Evaluation metrics (precision, recall, and F1 score) for height identification in simulated Pt-SAC STEM images. (**C**) Height identification in the experimental STEM image containing Pt-SACs, shown as a depth-resolved apparent Z-height map. (**D**) Statistics of dimer configurations revealing the huge discrepancy in experimental and simulated Pt-SAC STEM images. (**E**) Height identification across a focal series (defocus Δ*f*) showing consistent predictions from 0 to 9.6 nm. The confidence values represent the relative likelihood assigned to each depth hypothesis, listed in descending depth order (6, 4, 2, and 0 nm). Scale bars, 0.2 nm (A) and 0.5 nm [(C) and (E)].

The precision, recall, and F1 score metrics demonstrate reliable apparent *Z*-height classification of Pt atoms across the discrete depth classes of 0, 2, 4, and 6 nm. For the uniform am-C support, the CycleGAN-UNet model achieved reliable apparent Z-height classification, with average F1 scores of 0.96, 0.93, 0.94, and 0.93 for the 0-, 2-, 4-, and 6-nm classes, respectively (Fig. 4B). Given that real catalyst supports can vary in composition, thickness, crystallinity, and contamination level, they cannot be fully represented by a single uniform support approximation. To address this limitation, we constructed a support-specific simulation pipeline capable of generating matched training and validation datasets by varying the substrate type, local thickness variation, atomic species, atom density, pixel size, and noise/SNR level. For the am-C support with local thickness variation (fig. S16), the average F1 scores reached 0.98, 0.96, 0.95, and 0.95 for the 0-, 2-, 4-, and 6-nm classes, respectively (table S2). For the more complex case of a slightly tilted (∼2°) crystalline BP support (fig. S17), the corresponding average F1 scores were moderately reduced but remained high, with values of 0.95, 0.94, 0.92, and 0.90, respectively (table S3), despite the stronger structured background and possible channeling-related contrast.

These results confirm the overall robustness of the framework while also indicating that height classification under realistic support conditions still involves residual uncertainty (marked by arrows in figs. S16 and S17). Because apparent *Z*-height classification relies on subtle depth-dependent intensity attenuation and FWHM broadening, support-induced contrast, local thickness variation, contamination, and low SNR can perturb the apparent atomic profiles and introduce ambiguity between neighboring depth classes. To quantify this uncertainty, we further performed pooled, row-normalized confusion matrix analyses using support-specific simulated datasets (fig. S18 and tables S4 and S5). For the am-C support with local thickness variation, 97.64, 94.78, 94.88, and 93.33% of true 0-, 2-, 4-, and 6-nm atoms were correctly classified, respectively. For the more challenging slightly tilted (∼2°) crystalline BP support, the corresponding correct classification probabilities were 92.88, 92.91, 90.21, and 87.43%, respectively. The residual errors were mainly concentrated in adjacent-bin assignment and background assignment for weak-contrast atoms at larger depths (tables S4 and S5), indicating that the classification uncertainty is physically interpretable and quantitatively bounded. Under the relatively high-SNR (SNR = −1.23 dB) am-C condition with thickness variation, the correct classification probabilities remain above 93% for all four depth classes (table S4). Under the low-SNR contaminated condition (SNR = −4.44 dB), the corresponding probabilities decrease to 78.76, 87.46, 85.17, and 73.21% for true 0-, 2-, 4-, and 6-nm atoms, respectively (fig. S19 and table S6). The most pronounced degradation occurs in adjacent-bin assignment, especially true 0-nm atoms assigned to the 2-nm class, which increases from 2.36 to 21.24%, and background assignment for true 6-nm atoms, which increases from 5.93 to 18.75%. These results define the practical applicability boundary of the method: Regions with severe contamination, large thickness gradients, or background statistics outside the validated simulation domain should be interpreted cautiously or excluded from quantitative apparent Z-height analysis. The finer-bin validation (table S7) further suggests that the model retains sensitivity to sub-2-nm depth-dependent contrast variations under the present validation conditions, although the practical depth assignment remains a discrete apparent Z-height classification rather than continuous Z-coordinate reconstruction.

After validating the model on support-specific simulated datasets, we applied the trained inference model to experimental STEM images of high-density Pt ADCs to obtain depth-resolved atom maps, in which each detected atom is assigned to an apparent Z-height class relative to the calibrated top-surface focal reference (Fig. 4C and fig. S20). Analysis of height distributions and interatomic spacings reveals that more than 81% of atoms are monomers, whereas a substantial fraction of apparent dimers identified by conventional 2D projection–based analysis are reassigned as vertically separated atoms or depth-resolved configurations (Fig. 4D). Traditionally, shape fitting and Gaussian-based methods mainly identify single atoms, dimers, and trimers according to projected peak morphology and interpeak distance (*37*). By incorporating depth-dependent contrast information, the present framework reduces projection-induced overestimation of dimers by several folds and enables more reliable classification of local ADC configurations.

We further validated the physical consistency of the apparent *Z*-height assignment using through-focal STEM image series (*51*). Because the axial probe response is approximately symmetric around the focal plane (*50*), a single static image cannot uniquely determine the sign of defocus. In this work, the *Z* = 0 reference was therefore experimentally defined by a fast through-focal focusing procedure, in which representative near-surface Pt atoms first evolved from diffuse to sharp and then became diffuse again as the focus was further changed (fig. S21). The focal condition where these near-surface atoms exhibited the highest intensity and narrowest apparent profile was assigned as the top-surface focal reference. In the expanded through-focal validation (Fig. 4E and fig. S22), near-surface atoms reach maximum intensity and minimum FWHM close to the calibrated *Z* = 0 condition (atom 1 in fig. S22), whereas atoms predicted as deeper apparent *Z*-height classes become sharpest at larger positive defocus values (marked by arrows in Fig. 4E, atom 0 in fig. S22), consistent with the model predictions and illustrated by intensity line profiles shown in Fig. 1I. Accounting for focus variation effectively mitigates projection artifacts from 2D projection, reducing the incidence of unrealistically short interatomic distances (<0.2 nm) by ∼15% and increasing the overall atomic density by ∼30%, when blurred atoms are properly weighted (fig. S23). This improvement underscores the necessity of depth-aware correction in atomic-resolution analysis, enabling more reliable classification of vertically proximate atomic pairs unresolved in conventional 2D projections (Fig. 4C and fig. S24).

### Depth-aware identification of geminal sites and catalytic correlations

To further validate the power of our model in resolving complex atomic configurations beyond mononuclear sites, we extended our framework to the identification and interpretation of representative heterogeneous dimeric catalysts with well-defined structures. Among them, a notable example is the class of heterogeneous geminal-atom catalysts (GACs) reported by Lu *et al.* in 2023, where single-atom sites are paired with specific coordination and spatial proximity anchored on PCN. Demonstrated in *Nature* (*12*), their study revealed the pivotal role of geminal Cu-Cu (Cu_g_) dimers in promoting cross-coupling reactions via cooperative catalysis. The catalytic performance of Cu-GACs and Cu-SACs was systematically compared in C-N, C-O cross-coupling, and azide-alkyne cycloaddition reactions under identical Cu loadings, revealing substantial differences in activity. This disparity was attributed to the synergistic interaction between adjacent low-valent Cu atoms, which effectively lowers the activation barrier for oxidative addition and opens catalytic pathways inaccessible to isolated single atoms. However, such precise structural identification in their work required multimodal synchrotron-based spectroscopy and advanced theoretical modeling (*52*).

Our GAN-UNet framework directly resolved these geminal Cu-Cu dimers (metal-metal proximity of ∼4 Å) on PCN (Cu_g_/PCN) from ADF-STEM images (Fig. 5A), showcasing its capability to extract key structural motifs critical to catalytic performance. We further applied our model to Pt/PCN samples synthesized with different loadings, where the site-specific catalytic activity exhibits strong loading dependence (Fig. 5, B and C, and figs. S25 to S27). Like previous reports on Cu_g_/PCN, when the Pt/PCN loading is as low as 0.25 wt %, all Pt sites exist as isolated monomers; at a higher loading of 2.5 wt %, numerous geminal Pt sites can be observed. Impressively, the model successfully captured the structural transition from isolated Pt single atoms (Pt/PCN, 0.25 wt %) to Pt dimers (Pt_g_/PCN, 2.5 wt %), enabling a direct correlation between atomic configuration and catalytic performance. Under the same amount of Pt (2 mol %) conditions, the quinoline 2 product yields correlate with the metal content, increasing from 12% in the sample containing 0.25 wt % Pt, in which almost all the Pt sites are isolated monomers, to 84% over the catalyst with the highest content of geminal sites. Meanwhile, for isoquinoline 3 and 1*H*-indole 4, Pt_g_/PCN (2.5 wt %) also exhibits superior catalytic activity. To further evaluate catalytic performance, multiple parallel reaction batches were conducted under identical conditions using Pt/PCN with different loadings, respectively (Fig. 5D). The reactions were quenched at various time points to monitor and compare the reaction kinetics. These kinetic studies once again demonstrated the superior catalytic activity of Pt dimers over Pt-SACs. These results clearly demonstrate that our framework can reliably distinguish subtle but functionally critical differences in active site configurations, offering a powerful alternative to synchrotron-based methods. This approach facilitates structure-property prediction with atomic precision, thereby providing valuable guidance for the rational design and optimization of high-performance heterogeneous catalysts.

**Fig. 5.**
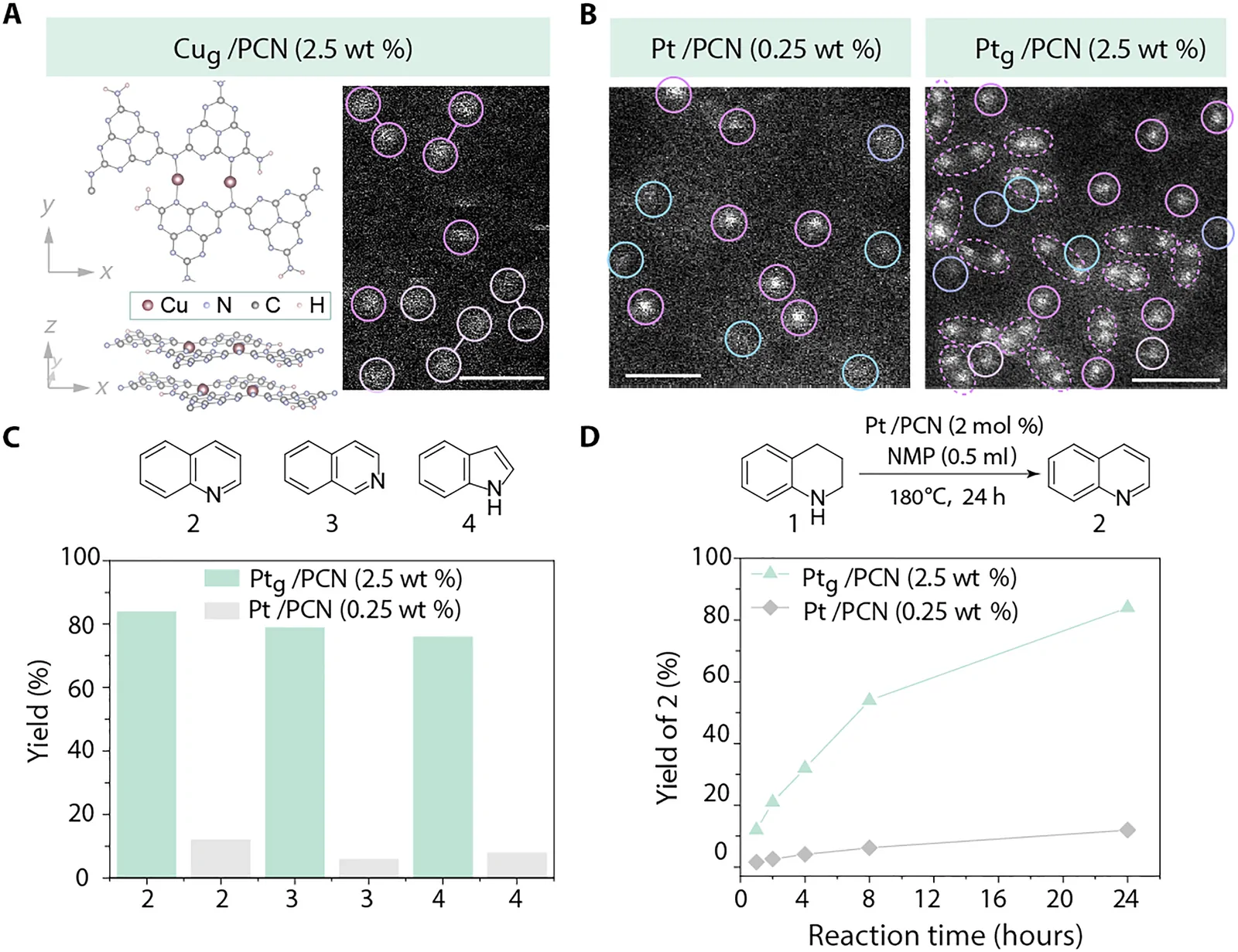
Catalytic performance and structural identification of metal geminal pairs on PCN. (**A**) Identification of Cu geminal pairs (Cu_g_ dimers) on PCN based on atom-height GAN-UNet model analysis. Two nearby Cu single atoms exhibiting identical height profiles are connected by solid lines, indicating the formation of Cu-Cu pairs. The corresponding atomic models are depicted on the left. (**B**) Height identification for low-loading Pt-SAC (0.25 wt %) and high-loading Pt geminal pairs (Pt_g_, 2.5 wt %) on PCN. The Pt dimers are highlighted by dashed circles. (**C**) Comparison of the catalytic performance for the dehydrogenation of 1,2,3,4-tetrahydroquinoline derivatives over Pt-SAC and Pt_g_ catalysts. Reaction conditions: 1,2,3,4-tetrahydroquinoline derivatives (0.1 mmol), [Pt] (2.0 mol %), NMP (0.5 ml), 180°C, 24 hours. (**D**) Kinetic profiles of quinoline formation showing superior reaction rates and yields of Pt_g_ over Pt-SAC. Scale bars, 0.5 nm (A) and 1 nm (B).

## DISCUSSION

Resolving vertical stacking allows us to exclude false dimers, thereby refining the true distribution of catalytically active monomers (*53*). This correction explains discrepancies often observed between apparent atomic and dimer densities and measured catalytic activity (*10*). Our model reveals a structural transition from isolated Pt monomers to geminal dimers as the loading increases, which directly correlates with the marked rise in quinoline yield (12% → 84%). We further observed that single-atom Pt sites still exist within the geminal Pt system, suggesting that a slight increase in Pt loading can effectively enrich the proportion of Pt_2_ dimers. Such quantitative linkage between configuration statistics and kinetic profiles demonstrates the power of our framework to precisely map 3D active site landscapes evolution at the atomic scale and provides a high-fidelity structural basis for understanding the nonlinear trends in activity-loading correlations. Beyond retrospective interpretation, this capability offers a predictive route to tune loading and dispersion toward optimal catalytic architectures. When assisted by our ML model, in situ liquid-phase ADF-STEM (*54*) could allow direct visualization and quantitative reconstruction of single-atom 3D dynamics within a fully reactive chemical environment at single-atom precision. This synergy enables the identification of metastable intermediates and their transformation kinetics, establishing atomic-scale correlations between dynamic site evolution and catalytic activity (*55*).

Overall, this framework provides a depth-aware route to extract more reliable active-site statistics from projected ADF-STEM images, enabling improved correlation between ADC configurations and catalytic performance. It should be noted, however, that the present framework provides discrete apparent Z-height classification rather than continuous 3D atomic reconstruction, and the practical depth assignment is defined by the validated bin size and imaging conditions. Its reliability also depends on the match between the simulated training domain and the experimental system. For broader application, support-specific simulations and experimental calibration should be incorporated before applying the model to new ADC systems. In practice, this requires constructing representative structural models of the target support, including realistic thickness or contrast variations.

## MATERIALS AND METHODS

### Synthesis of ADC on substrates

The preparation of SACs on carbon support followed a previously reported two-step annealing protocol (*17*), whereas SACs on BP were prepared via atomic layer deposition (*56*). Synthesis of Pt_g_/PCN:H_2_PtCl_6_·6H_2_O (Merck, ACS reagent, ≥37.50% Pt basis) and exfoliated PCN were dispersed in 200 ml of formamide (Sigma-Aldrich, ACS reagent, ≥99.5%), sonicated for 10 min, and then stirred in an oil bath (120°C) for 12 hours, following centrifugation and washing thoroughly by ethanol (Sigma-Aldrich, ACS reagent, ≥99.5%) several times. The oven-dried powder (80°C) was subsequently heated to 500°C with a heating rate of 2°C min^−1^ and kept for 5 hours with the protection of N_2_ flow. Pt/PCN with Pt contents of 0.25 and 2.5 wt % were prepared using 0.3 and 3.0 wt % Pt feeding, respectively.

### STEM characterization, simulation, and image processing

ADF-STEM imaging was performed on an aberration-corrected JEOL NEOARM200F, equipped with a cold field emission gun. The accelerating voltage was 80 kV to reduce beam damage. The convergence semiangle of the probe was ∼32 mrad. The collection angle of STEM images was between 80 and 200 mrad. To generate training data for the GAN-UNet model, simulated STEM images were produced using the abTEM Python library (*57*), assuming an aberration-free probe. The accelerating voltage, convergence semiangle, and collection angle were in line with the experiments. To ensure consistency with experimental images at varying magnifications, the pixel size of the simulations was set within the range of 0.1 to 0.3 Å.

Atomic models for simulation were constructed with and without am-C. The uniform am-C support was generated with a density of 2.16 g/cm^3^. To address the effect of support-dependent contrast, support-specific simulated datasets were constructed, including am-C with local thickness variation and slightly tilted crystalline BP supports.

To match the atom densities observed in experimental conditions, the atom density was set between 0.43 atom/nm^3^ (2.8 atom/nm^2^) and 1.57 atom/nm^3^ (10.3 atom/nm^2^). To emulate the presence of atomic assemblies, such as dimers and trimers, the minimum projected interatomic distance was constrained to 0.8 Å. The depth range of Pt was set to 6 nm, with atoms either randomly distributed or specifically positioned at heights of 0, 2, 4, and 6 nm. The Pt atom image was assumed to be rotationally symmetric. The peak intensity and FWHM were refined using a 2D Gaussian fit applied to 16 × 16 pixel patches of the image, a region enclosing the total Pt.

The U-Net training dataset was constructed from CycleGAN-enhanced simulated STEM images with pixel-wise annotations of atomic positions and apparent *Z*-height classes. The simulated images and corresponding ground-truth labels were cropped into approximately 1500 paired 256 × 256 image patches for training. Because each patch contained multiple atomically dispersed metal sites, the training dataset included more than 150,000 labeled single-atom positions in total. To improve robustness and reduce overfitting, the simulated data covered a range of experimentally relevant conditions, including different pixel sizes, atom densities, SNR/background levels, and contrast fluctuations. During U-Net training, stochastic data augmentation was applied, including resizing, random cropping, flipping, sharpening/smoothing, brightness and contrast perturbation, and noise variation. These strategies were used to improve the robustness of atom localization and apparent *Z*-height classification across different support conditions.

### CycleGAN training

The CycleGAN network was trained using a dataset comprising 1500 simulated cropped images with varying pixel sizes and 1500 experimental cropped images featuring different substrates and different ADCs. To better align the image styles of simulated images with experimental conditions, Gaussian noise, Poisson noise, and local variance noise were added to the simulated images before training. These noise-augmented simulated images were designated as domain A, whereas the experimental images were set as domain B. The CycleGAN model was trained to learn bidirectional mappings between these two domains. Training was conducted using a batch size of 42 over 68 epochs. The initial learning rate was set to 2 × 10^−4^ and was linearly decayed to zero starting from the midpoint of training. The Adam optimizer was used to update network parameters throughout the training process.

### UNet training

We used a UNet-based convolutional neural network for ADC detection tasks, consisting of an encoder-decoder architecture with skip connections. Each stage in both the encoder and decoder comprises a custom-designed convolutional block that includes two convolutional layers with varying kernel sizes (3 × 3 and 5 × 5), followed by batch normalization and ReLU activations. This design allows the model to capture multiscale contextual information while maintaining spatial resolution. Dropout regularization with a probability of 0.5 is optionally included in the convolutional blocks to mitigate overfitting. The encoder path consists of four levels, with feature dimensions increasing from 64 to 512 through successive convolutional blocks, each followed by a 2 × 2 max pooling layer to downsample the spatial resolution. A bottleneck layer with 1024 feature channels is applied at the bottom of the U-shaped architecture. In the decoder path, transposed convolutional layers are used to upsample the feature maps, each followed by a corresponding convolutional block. At each decoding stage, the upsampled feature maps are concatenated with the corresponding encoder feature maps via skip connections, enabling the network to recover spatial details lost during downsampling. Last, a 1 × 1 convolutional layer is applied to reduce the feature dimension to the number of target classes. The UNet model was trained on a dataset consisting of 1500 cropped images generated from CycleGAN-enhanced simulated data, along with their corresponding labels. Training was performed with a batch size of 16 over 30 epochs. A StepLR learning rate scheduler was used, starting with an initial learning rate of 0.01, with a step size of 100 and a decay factor of 0.1. The Adam optimizer was used for model optimization. To improve segmentation accuracy and robustness, a composite loss function was adopted, combining cross-entropy loss, Dice loss, and Focal loss.
