# Supplementary material for: Deep learning–assisted electron microscopy for apparent Z-height mapping of atomically dispersed catalysts
Source: Sci Adv. 2026 Nov 13;12(46):eaef7673. doi: 10.1126/sciadv.aef7673 (PMC13763925; doi:10.1126/sciadv.aef7673)
Supplement: Supplementary file 1 [file sciadv.aef7673_sm.pdf]

Science Advances
AAAS

Supplementary Materials for

Deep learning–assisted electron microscopy for apparent Z-height mapping of atomically dispersed catalysts

Xiaocang Han *et al.*

Corresponding author: Yanhui Hong, hongyanhui@dp.tech; Zezhong Zhang, zhangzz@aisi.ac.cn; Jiong Lu, chmluj@nus.edu.sg; Xiaoxu Zhao, xiaoxuzhao@pku.edu.cn

Sci. Adv. **12**, eaef7673 (2026)
DOI: 10.1126/sciadv.aef7673

This PDF file includes:

Figs. S1 to S27
Tables S1 to S7

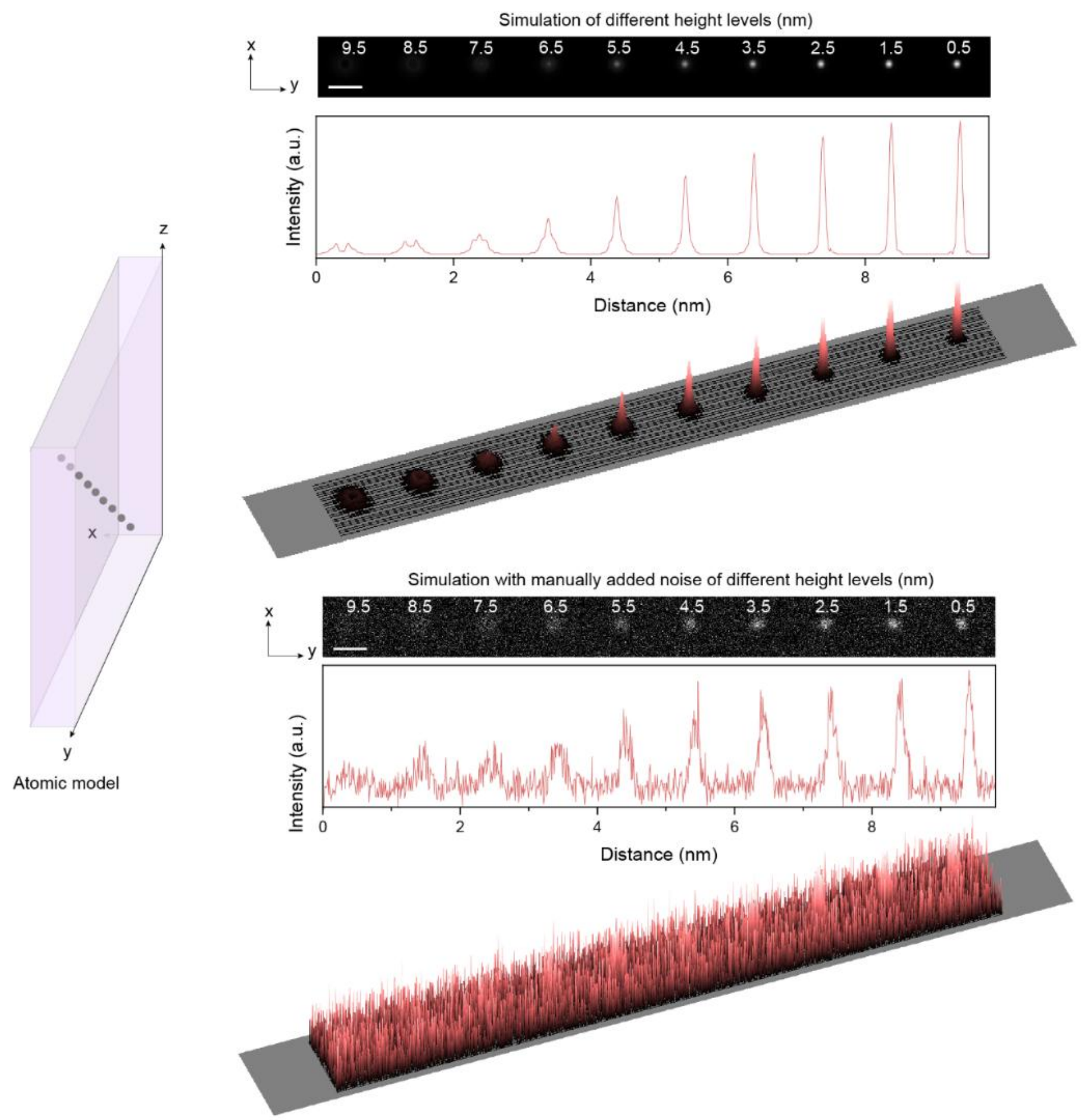

Figure S1 Intensity variations caused by different atomic heights. The left panel shows the atomic model of Pt atoms positioned at varying heights. The right panel illustrates the simulated annular dark-field scanning transmission electron microscopy (ADF-STEM) images, the corresponding intensity line profiles, and three-dimensional surface plots of intensity variations, both without and with added noise (Gaussian, Poisson, and local variance noise), respectively. In this simplified simulation, the support was excluded to isolate the height-/defocus-dependent contrast variation of Pt atoms. Under noisy conditions, the contrast difference between neighboring 1 nm height levels becomes less distinguishable, indicating increased uncertainty for 1 nm-scale height discrimination. Scale bars, 0.5 nm

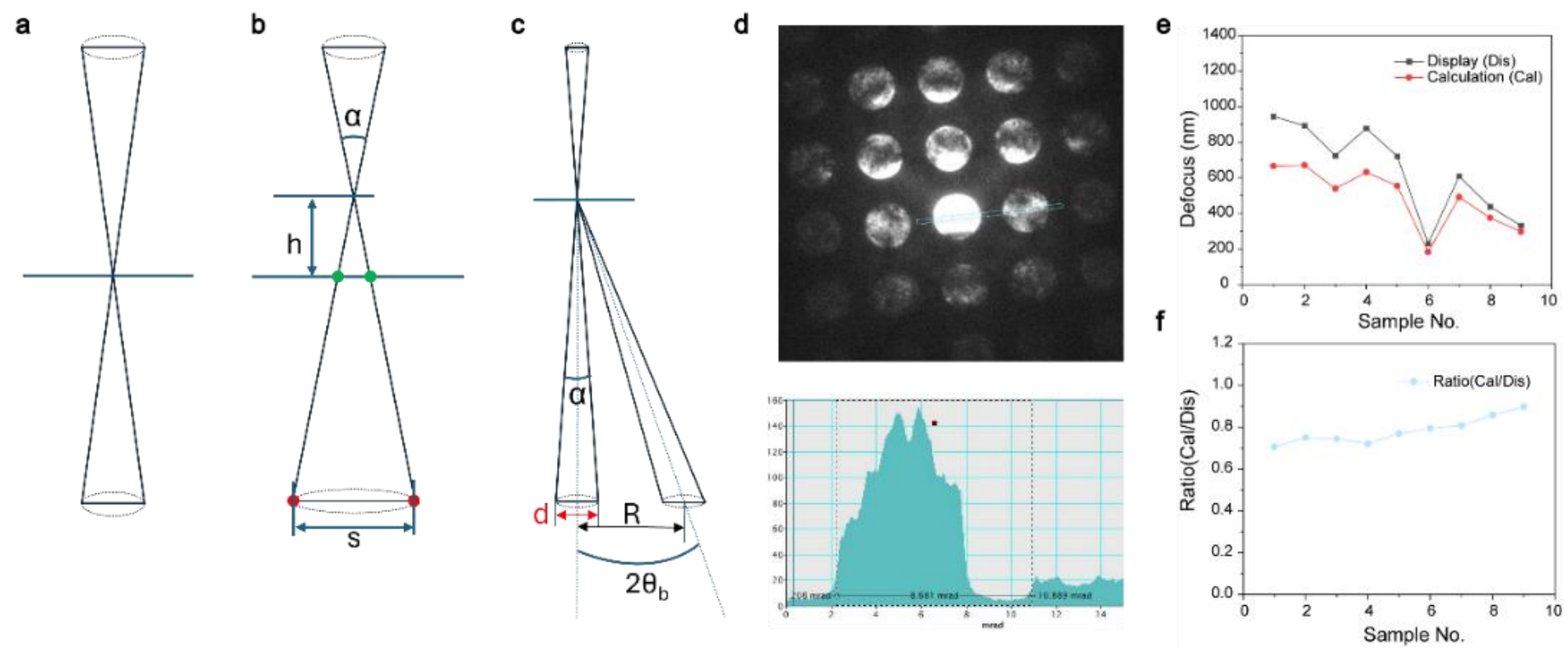

Figure S2 Schematic illustration and experimental validation of convergence-angle and defocus calibration. a, Schematic of the in-focus condition. b, Defocused condition, where h denotes the true defocus distance, α the convergence angle, and s the separation between two points that appear in the image plane under defocus. c, Geometric construction for determining the convergence full angle α, where d is the diameter of the direct beam, R the distance between the diffracted spot and the direct beam, and θ_b the Bragg angle. d, Experimental diffraction pattern of Si [100] used for α calibration, with the corresponding intensity profile showing the measured separation R ($R = 8.681$ mrad) between the (020) diffracted spot and the direct beam. e, Displayed (Dis) and calculated (Cal) defocus values for nine measurement sets. f, Corresponding ratios of calculated to displayed values (Cal/Dis), demonstrating an average agreement between the true 0.78 nm defocus and 1.0 nm display calibration.

During the experiment, defocus was introduced by adjusting the objective lens from the in-focus condition (Fig. S2a) to the defocused condition (Fig. S2b). The corresponding defocus value read from the microscope was then compared with the calculated h to determine the true defocus calibration. The relationship between these parameters is given by Eq. (1):

$$h = \frac{s}{2\tan(\alpha/2)} \qquad (1)$$

where h denotes the true defocus distance, α the convergence angle, and s the distance between the two particles, which can be measured in scan mode.

To determine the actual convergence angle α (Fig. S2c), we used a standard Si sample for calibration. For Si along the [100] zone axis, the interplanar spacing of the (020) plane is $d_{(020)}$ = 0.2715 nm, giving a Bragg angle of θ_b = 4.6225 mrad. The convergence full angle α can be determined from the diffraction geometry according to Eq. (2):

$$\alpha = \frac{d \times 2\theta_b}{R} \qquad (2)$$

where R is the distance between the (020) diffracted spot and the direct beam, and d is the diameter of the direct-beam disc.

As measured experimentally, the distance between the (020) diffracted spot and the direct beam is 8.681 mrad (Fig. S2d), yielding a convergence full angle of α = 49.95 mrad. All measurements were performed under STD focus conditions. Defocus calibration was subsequently performed by recording both the actual sample size (s) and the corresponding defocus values displayed by the microscope. The results indicate that a displayed defocus of 1.0 nm corresponds to a true defocus of 0.78 nm after calibration.

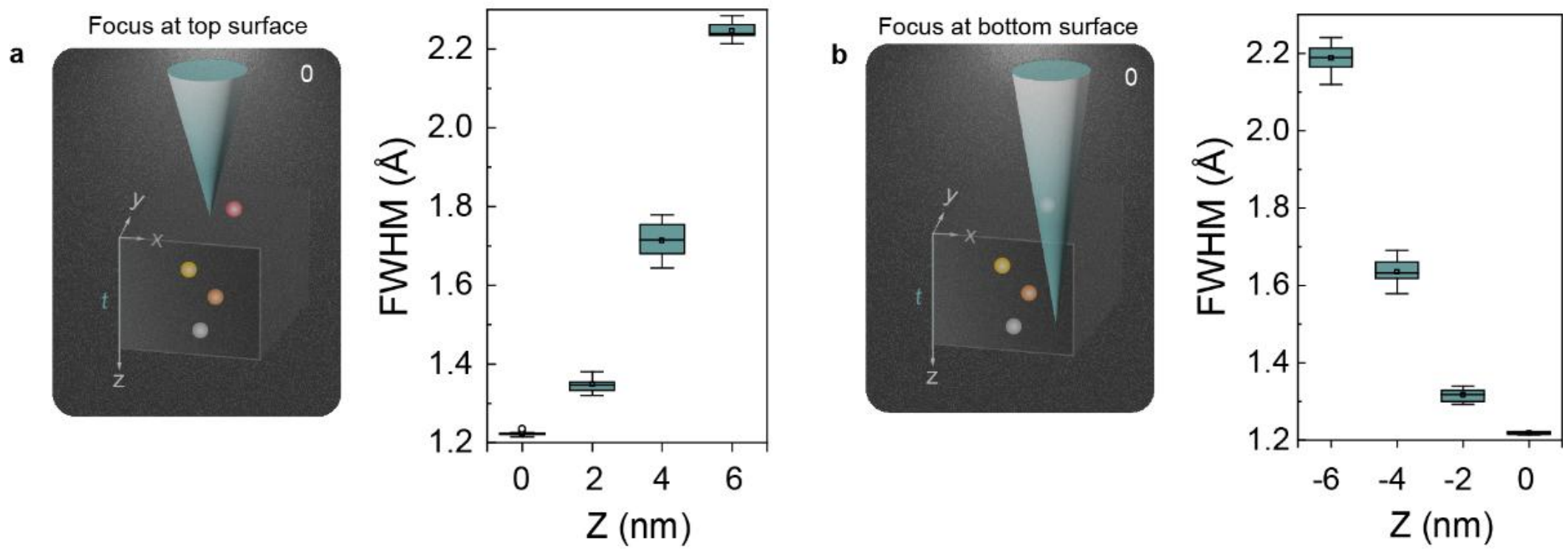

Figure S3 The full width at half maximum (FWHM) variations caused by changes in the focal plane. a, b, Electron beam focused on the top and bottom planes, respectively. The corresponding box plots show the FWHM of Pt atomic blobs as a function of depth (Z-height), with a step size of 2 nm.

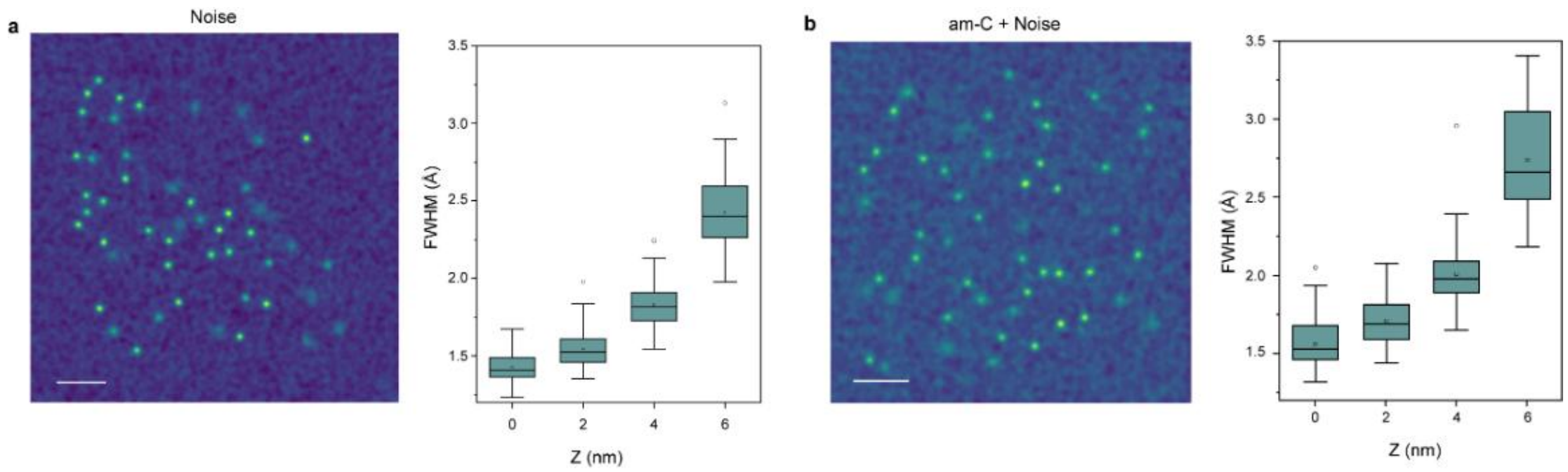

Figure S4 The FWHM variations caused by substrate and background noise. a, b, Simulated ADF-STEM images of Pt atoms at different heights, with and without amorphous carbon (am-C), in the presence of added noise (Gaussian, Poisson, and local variance noise). The corresponding box plots depicted on the right panels show the FWHM of Pt atom blobs as a function of depth (Z) height, with a step size of 2 nm. Scale bars, 1 nm.

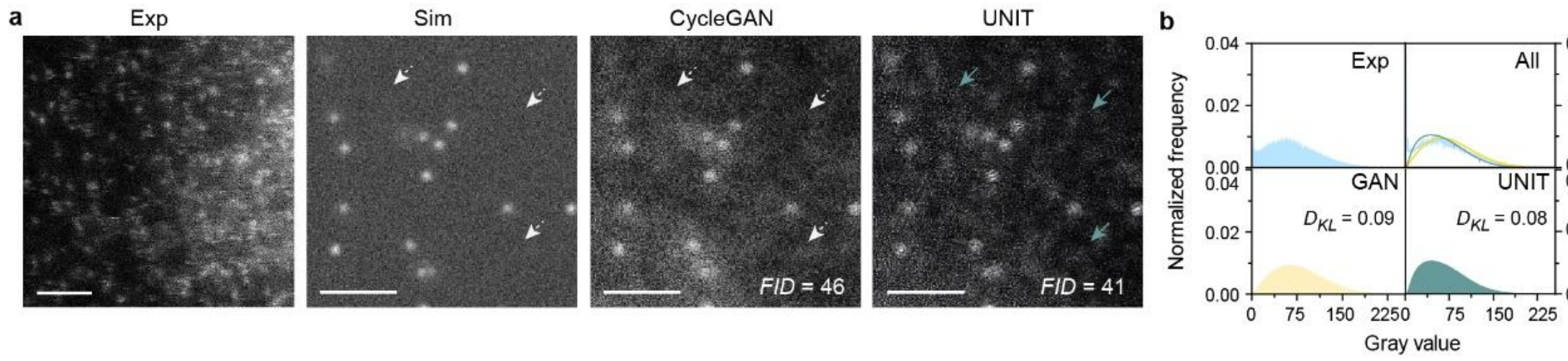

Figure S5 Comparison of generated training data images via CycleGAN and unsupervised image-to-image translation (UNIT). Scale bars, 1 nm.

a, The experimental (*Exp*), noise-added simulated (*Sim*), CycleGAN-generated, and UNIT-generated images. Arrows highlight regions in the UNIT-generated image where there is an increase in atoms with weak contrast compared to the CycleGAN-generated image. b, The corresponding histograms display the normalized frequency of gray values for the Exp, CycleGAN, and UNIT images. The Kullback-Leibler divergence (D_{KL}) values are provided for each comparison, combined with the Fréchet inception distance (FID) values for CycleGAN (FID = 46) and UNIT (FID = 41), indicating comparable similarity between the CycleGAN and UNIT-generated images to the experimental data. However, when learning from the continuously changing backgrounds (Left *Exp*), UNIT tends to generate additional false atomic positions with weak contrast, limiting its generalizability.

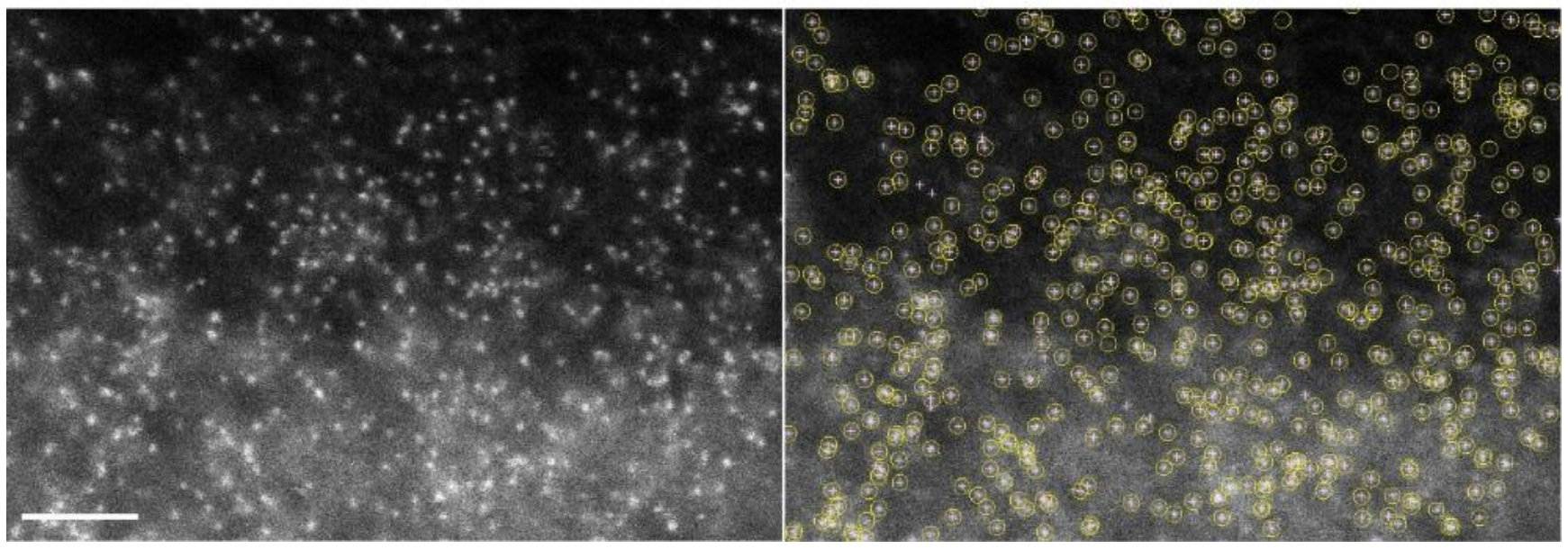

Figure S6 Raw STEM images of Pt on am-C (left), and corresponding human labeling and model predictions (right). Crosses and circles denote the human labeling and model predictions, respectively. Scale bar, 2 nm.

The atoms recognized by both the human and the model are counted as true positives (TP, 523), while atoms identified only by the model are false positives (FP, 29), and atoms missed by the model are false negatives (FN, 30).
To quantitatively evaluate the model's performance, precision, recall, and F1 score were calculated according to Eqs. (3)–(5):

$$\text{Precision} = \frac{\text{TP}}{\text{TP} + \text{FP}} \quad (3)$$

$$\text{Recall} = \frac{\text{TP}}{\text{TP} + \text{FN}} \quad (4)$$

$$\text{F1} = 2 \times \frac{\text{Precision} \times \text{Recall}}{\text{Precision} + \text{Recall}} \quad (5)$$

Precision reflects the model's ability to avoid false detections, recall evaluates its capability to identify real atoms, and the F1 score provides a balanced assessment of both metrics. The calculated values are summarized in Fig. 3D. However, due to the low contrast of atoms located away from the focal plane, accurate performance evaluation remains challenging. Therefore, a weighted evaluation strategy that accounts for weak-contrast atoms was further adopted in the apparent Z-height classification analysis.

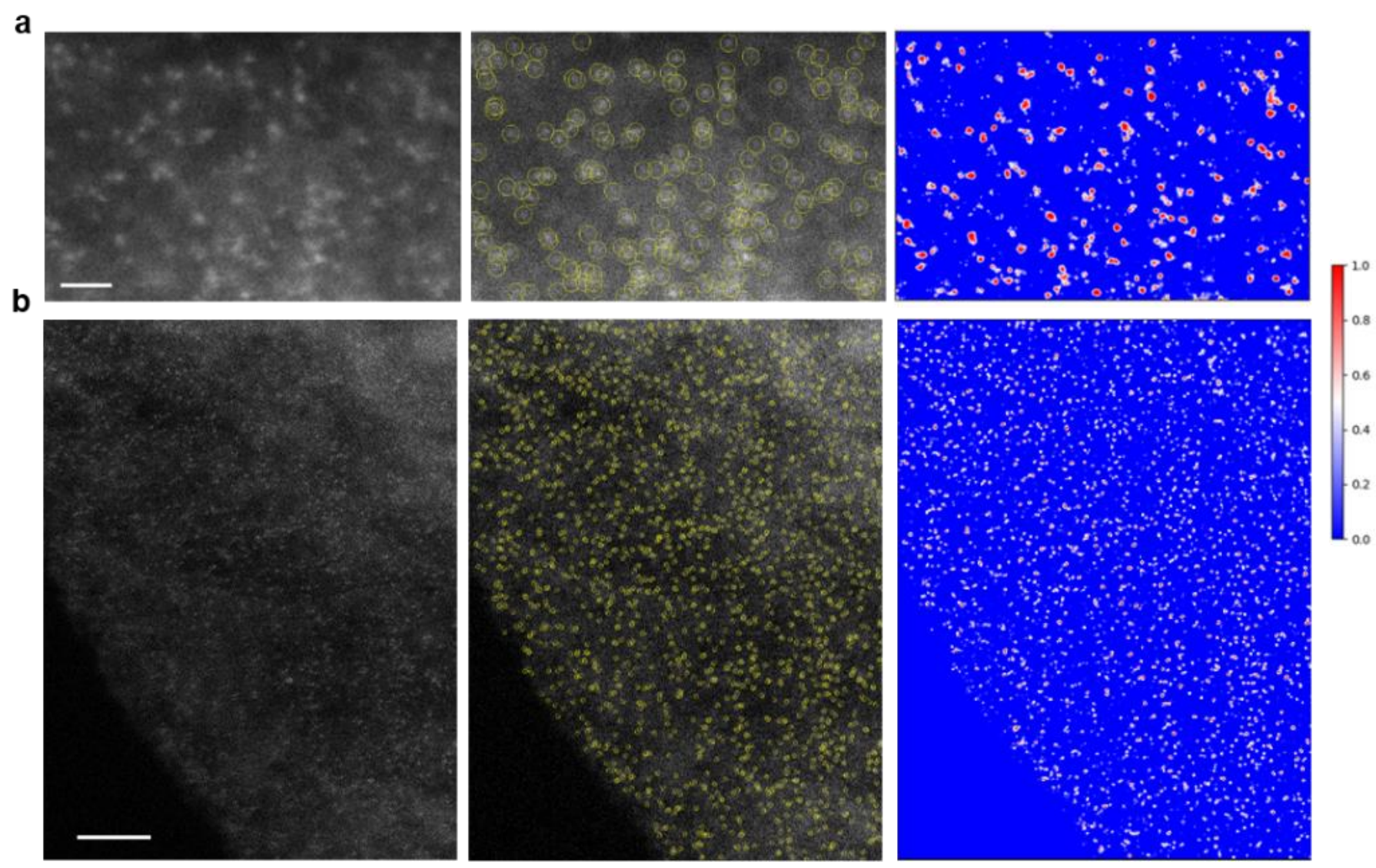

Figure S7 Automated detection and identification of single Pt atoms in experimental STEM images using GAN-U-Net-based models. a, b, Atom detection under conditions of (a) contamination and (b) relatively low magnification with a low signal-to-noise ratio (SNR). Scale bars: 1 nm (a), 5 nm (b).

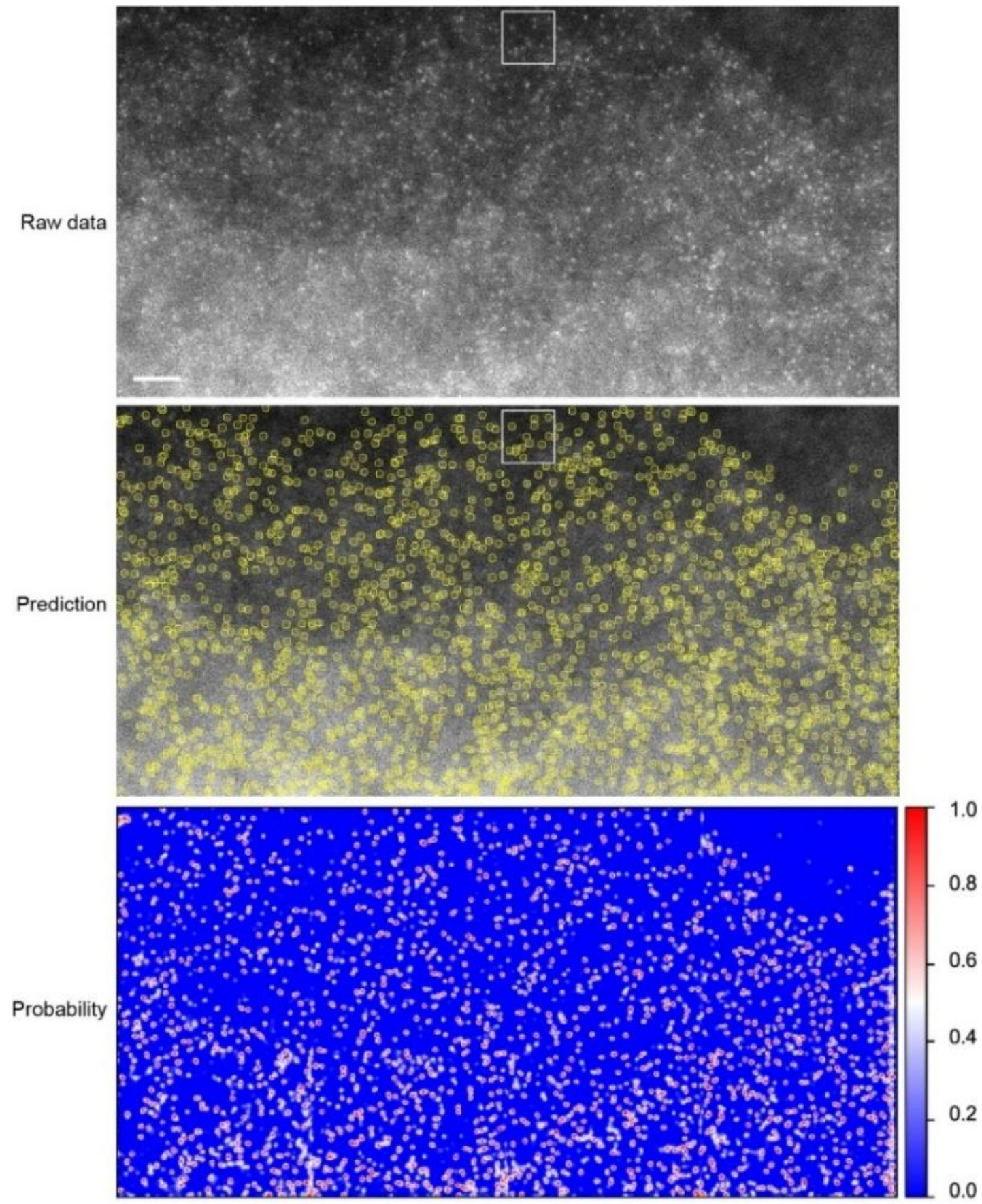

Figure S8 Automated detection and identification of single Pt atoms in low SNR experimental STEM images using GAN-U-Net-based models. The region marked by the white frame corresponds to the STEM image shown in Fig. 3G. Scale bar, 2 nm.

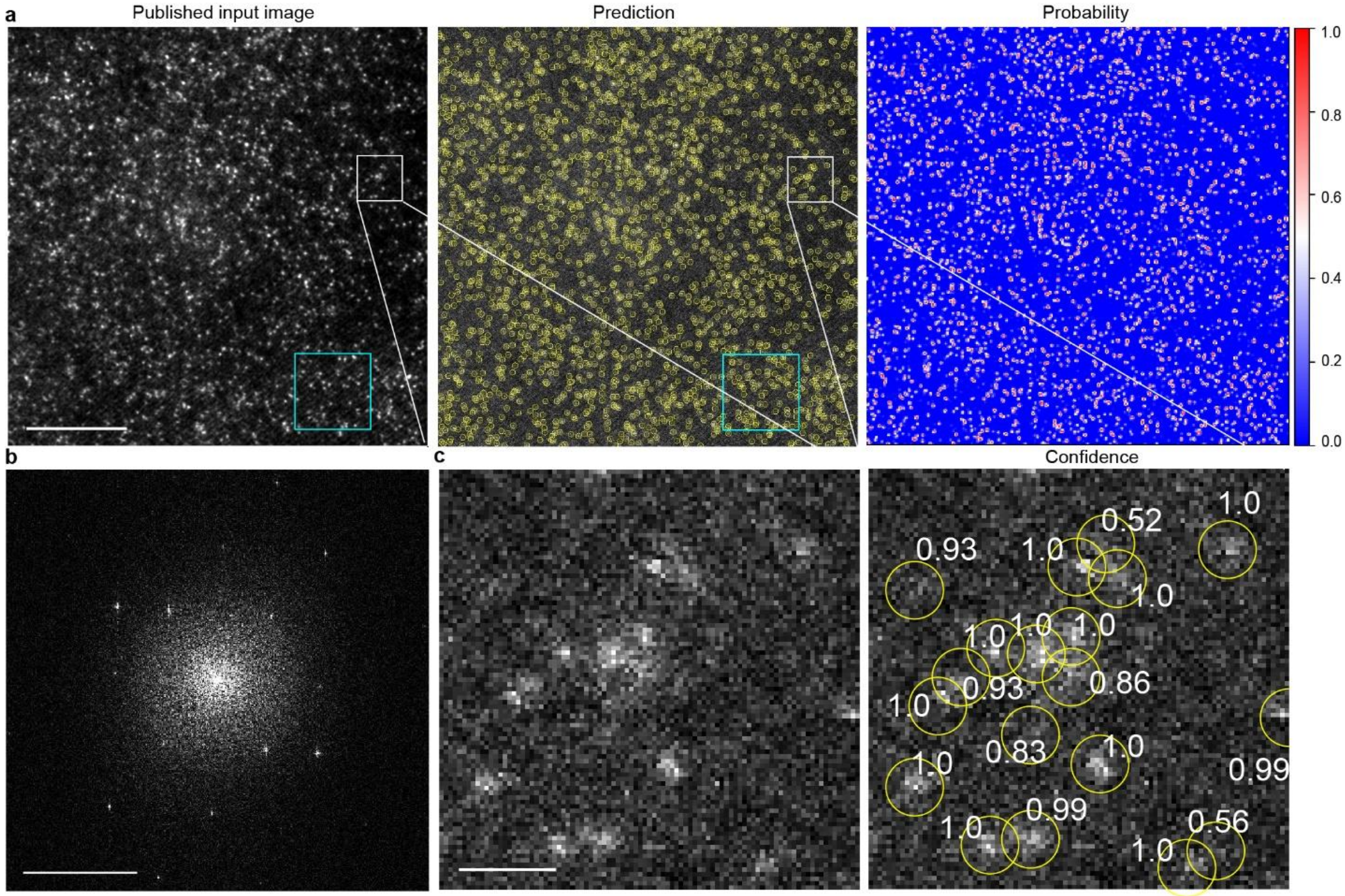

Figure S9 Automated detection and identification of single Pt atoms on crystalline black phosphorus (BP) in experimental STEM images using GAN–U-Net–based models. a, The prediction results. b, Corresponding FFT pattern confirming the crystalline nature of BP. c, Enlarged view showing the predicted atomic positions. The cyan-framed region corresponds to the STEM image shown in Fig. 3H. The input STEM image was adapted from supplementary fig. S11b of Chen et al. (*56*), with permission from Wiley. Copyright 2021 Wiley-VCH GmbH. The image was cropped and contrast-adjusted for visualization. Prediction overlays and the probability map were generated in this study. Scale bars: 5 nm (a), 5 nm^{-1} (b), 0.5 nm (c).

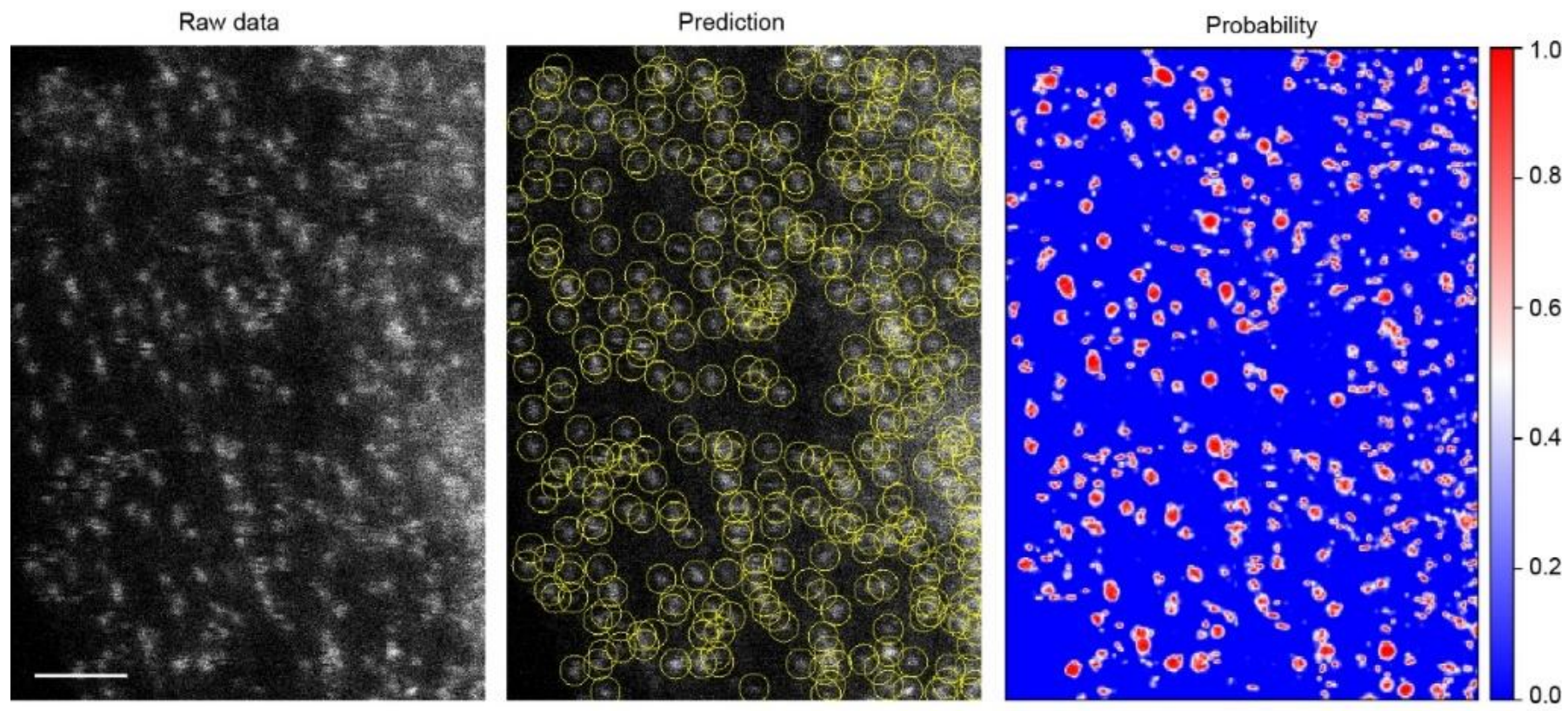

Figure S10 Automated detection and identification of single Pd atoms on am-C in experimental STEM images using GAN-U-Net-based models. Scale bar, 1 nm.

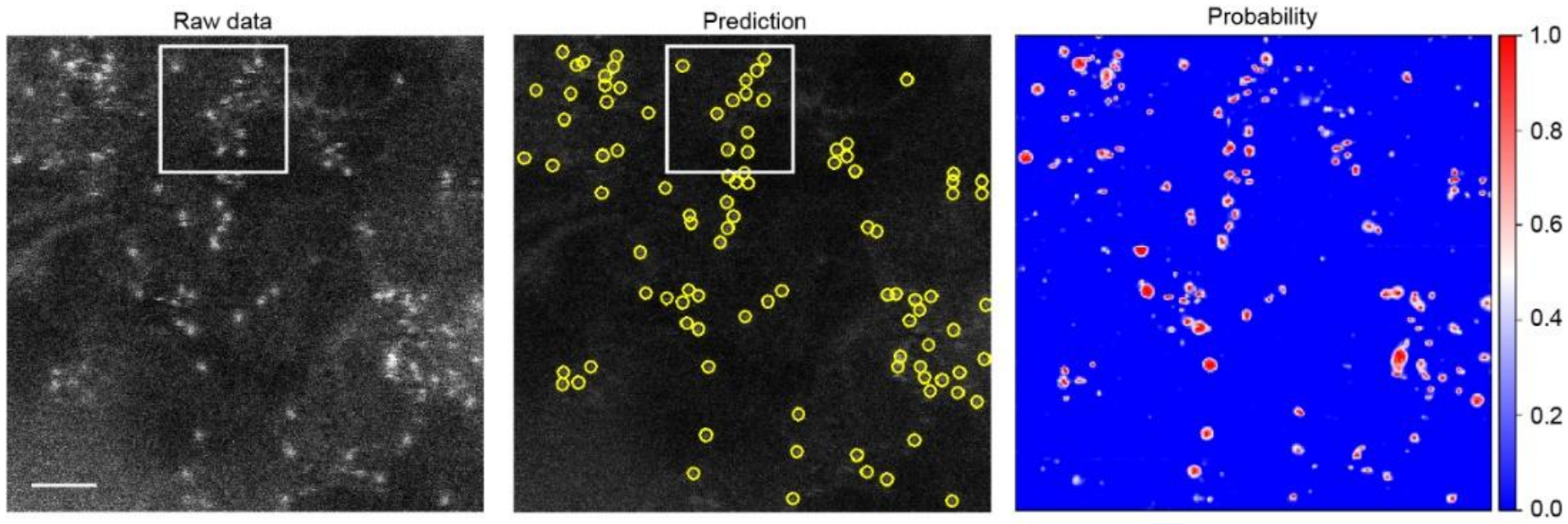

Figure S11 Automated detection and identification of single Fe atoms on carbon support in experimental STEM images using GAN-U-Net-based models. The region marked by the white frame corresponds to the STEM image shown in Fig. 3I. Scale bar, 1 nm.

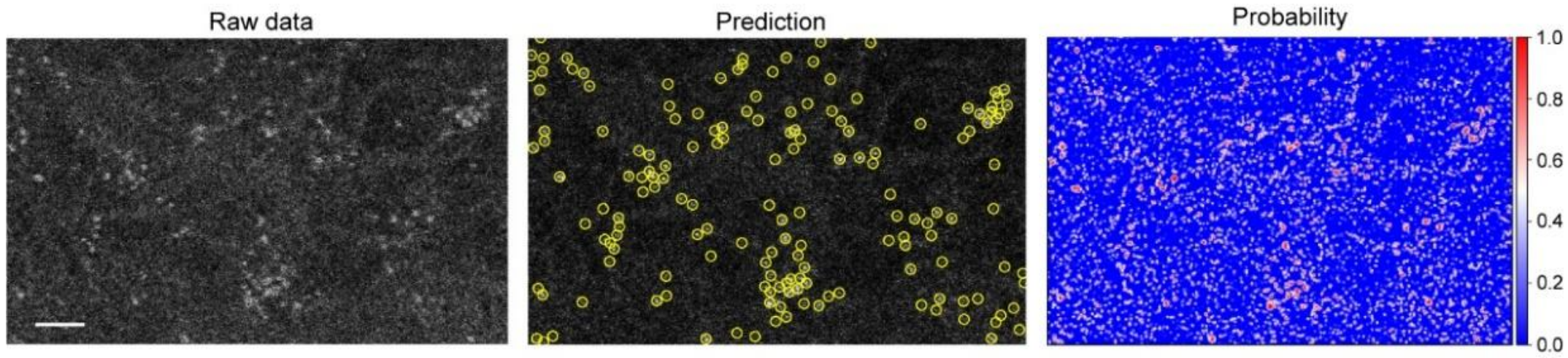

Figure S12 Automated detection and identification of single Mn atoms on carbon support in experimental STEM images using GAN-U-Net-based models. Scale bar, 1 nm.

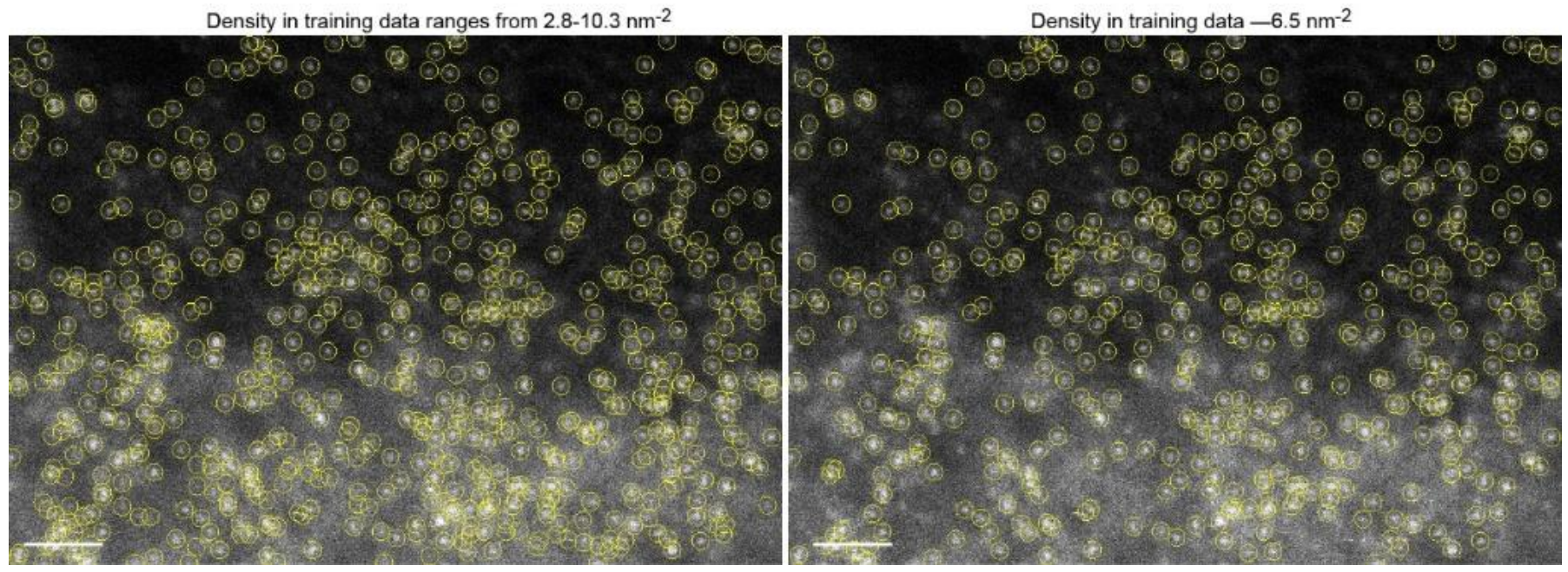

Figure S13 Atomic detection performance across different atomic densities in the training datasets. The atomic densities range from 2.8 to 10.3 atoms per nm^2, or single-atom density (6.5 nm^{-2}), demonstrating the impact of density variations on the model's performance. Scale bars, 2 nm.

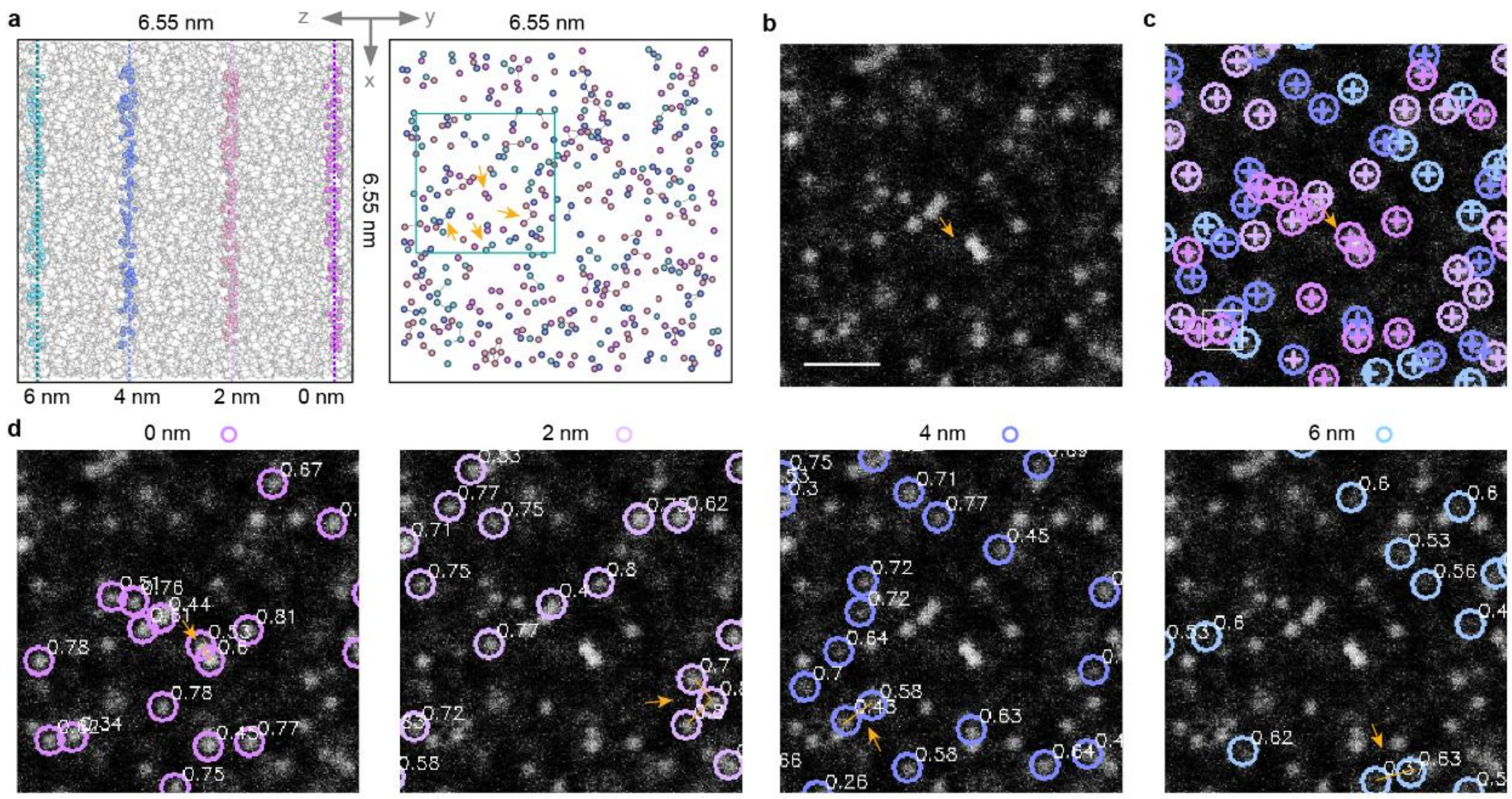

Figure S14 Automated apparent Z-height detection and identification of Pt atoms on uniform am-C support in simulated images using the GAN–UNet-based model. a, Atomic models of Pt atoms placed at different apparent Z-height positions on am-C support, used for ADF-STEM image simulation. The am-C support model in the top-view image (right panel) was removed to more clearly show the positions of the Pt atoms. b, Simulated ADF-STEM image of Pt single atoms on the am-C support with CycleGAN-generated experimental-like noise. c, Predicted apparent Z-height classes of Pt single atoms in the same region as shown in (b), with colored circles indicating model predictions and crosses indicating ground-truth Pt positions. d, Separated color maps showing Pt atoms assigned to different apparent Z-height classes. Dimers and trimers are linked by bonds and denoted by arrows, showing excellent agreement with the ground truth. The white box in (c) highlights a representative misclassification between the 0 and 2 nm apparent Z-height classes. Scale bar, 0.5 nm.

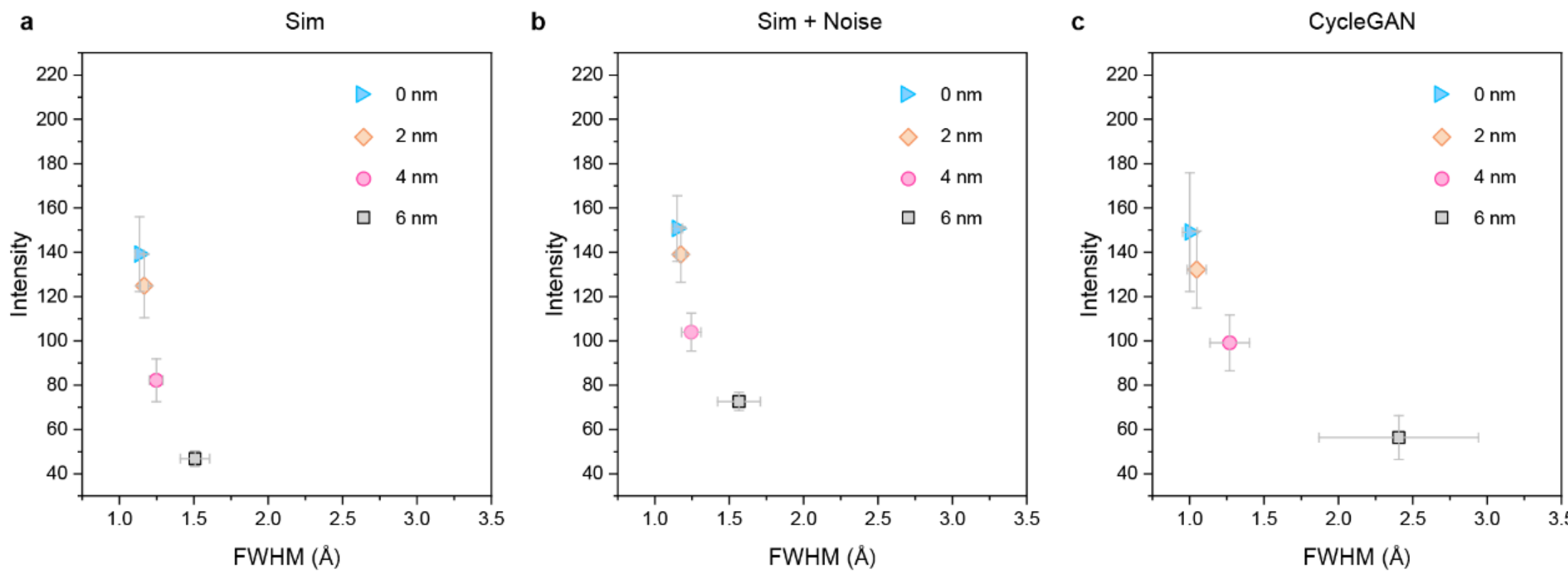

Figure S15 Class-averaged intensity-FWHM statistics before and after CycleGAN translation. a-c, Class-averaged peak intensity and FWHM of Pt atomic blobs labelled at apparent Z-heights of 0, 2, 4, and 6 nm in (a) the original simulations, (b) simulations with manually added noise (Poisson and Gaussian), and (c) CycleGAN-translated pseudo-experimental images. Horizontal and vertical error bars represent ±1 standard deviation of the extracted FWHM and intensity, respectively. The class-averaged results further show that CycleGAN preserves the depth-dependent ordering of the physical descriptors while increasing the statistical dispersion expected for pseudo-experimental images.

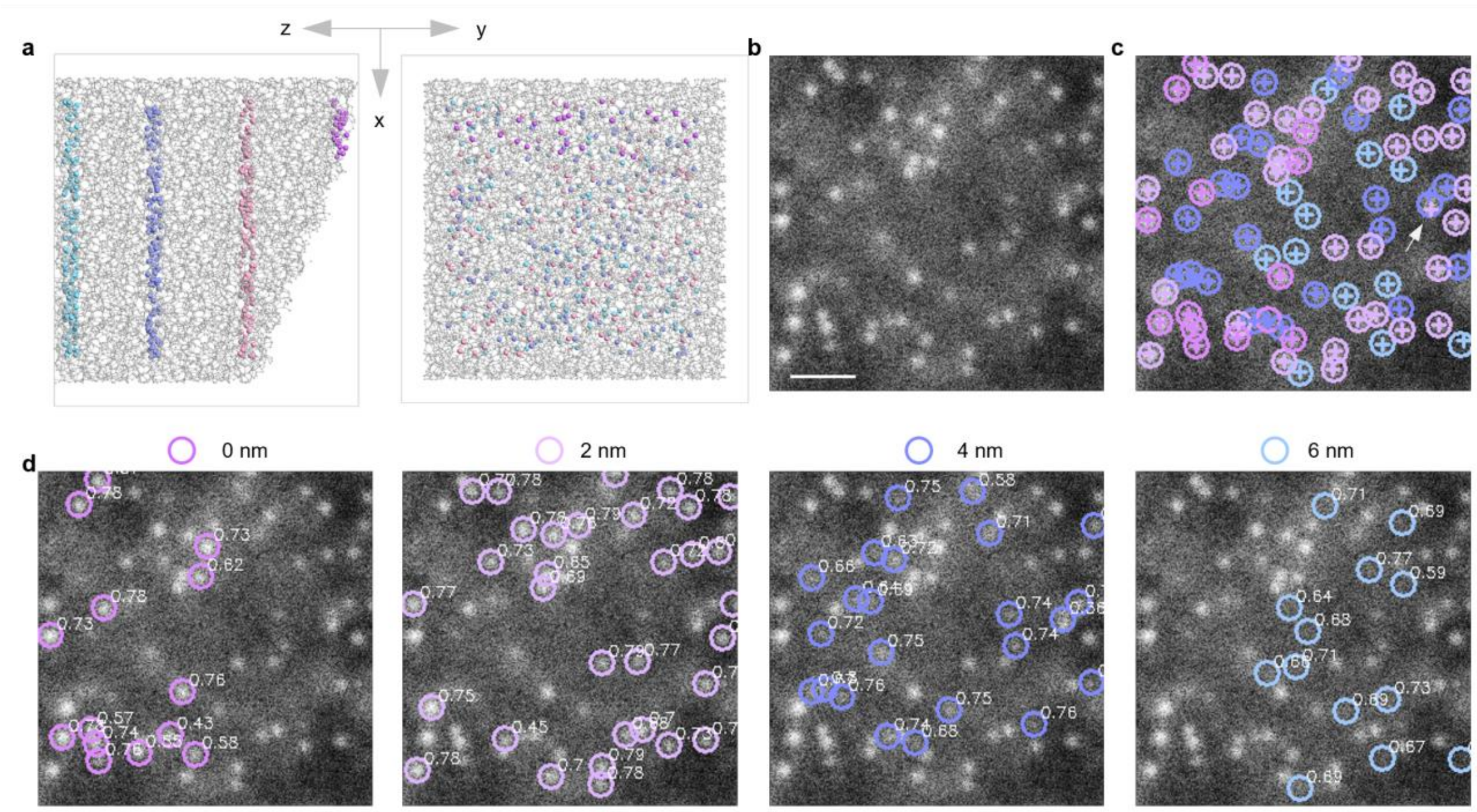

Figure S16 Automated apparent Z-height detection and identification of Pt atoms on am-C support with local thickness variation in simulated images using the GAN–UNet-based model. a, Atomic models of Pt atoms placed at different apparent Z-height positions on am-C support with local thickness variation, used for ADF-STEM image simulation. b, Simulated ADF-STEM image of Pt single atoms on the am-C support with CycleGAN-generated experimental-like noise. c, Predicted apparent Z-height classes of Pt single atoms in the same region as shown in (b), with colored circles indicating model predictions and crosses indicating ground-truth Pt positions. d, Separated color maps showing Pt atoms assigned to different apparent Z-height classes. Scale bar, 0.5 nm.

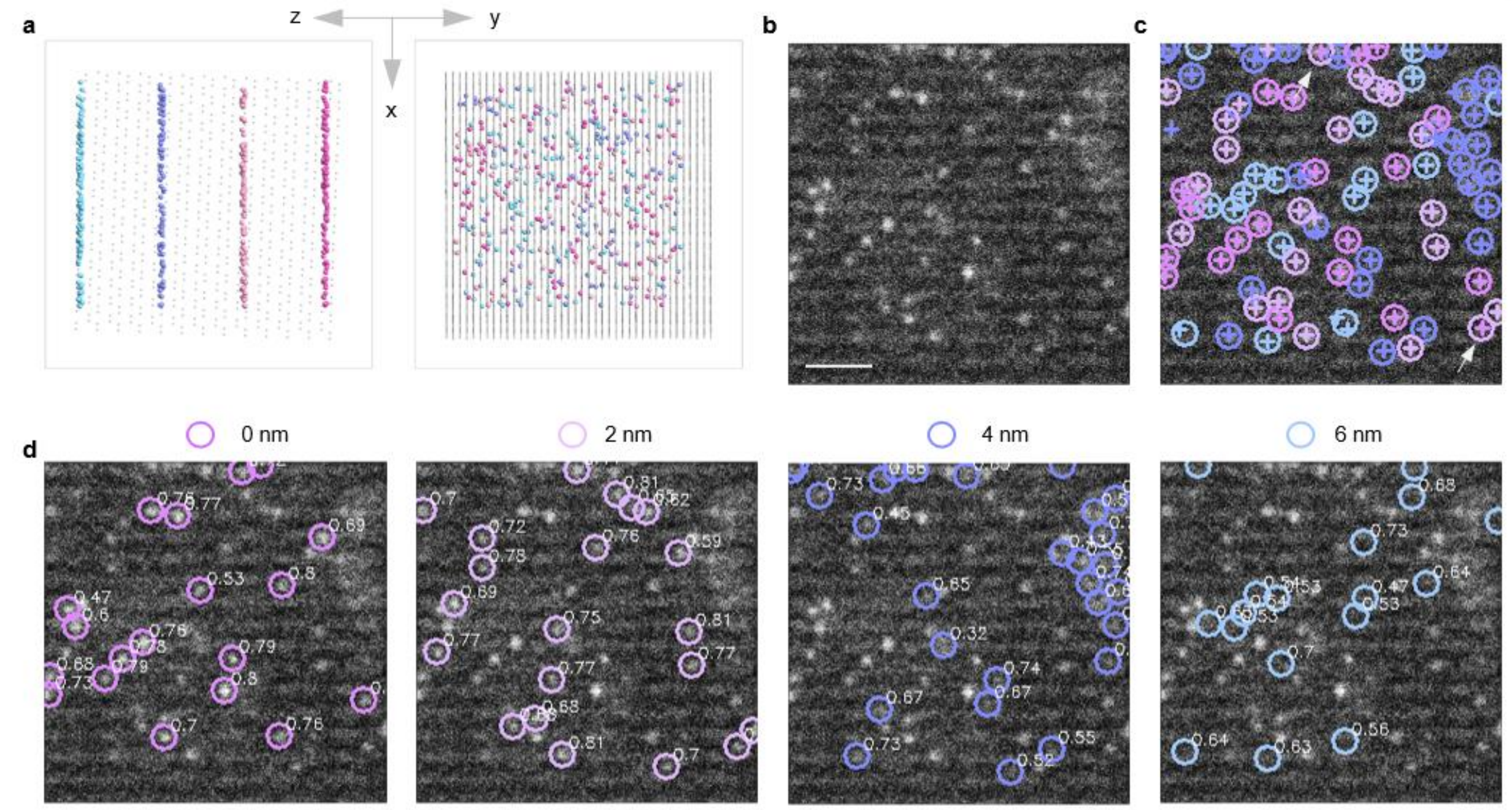

Figure S17 Automated apparent Z-height detection and identification of Pt atoms on a slightly tilted crystalline BP support in simulated images. a, Atomic model of Pt single atoms supported on a slightly tilted (~2°) crystalline BP substrate, used for ADF-STEM image simulation. b, Simulated ADF-STEM image of Pt single atoms on the slightly tilted crystalline BP support with CycleGAN-generated experimental-like noise. c, Predicted apparent Z-height classes of Pt single atoms in the same region as shown in (b), with colored circles indicating model predictions and crosses indicating ground-truth Pt positions. d, Separated color maps showing Pt single atoms assigned to different apparent Z-height classes. Scale bar, 0.5 nm.

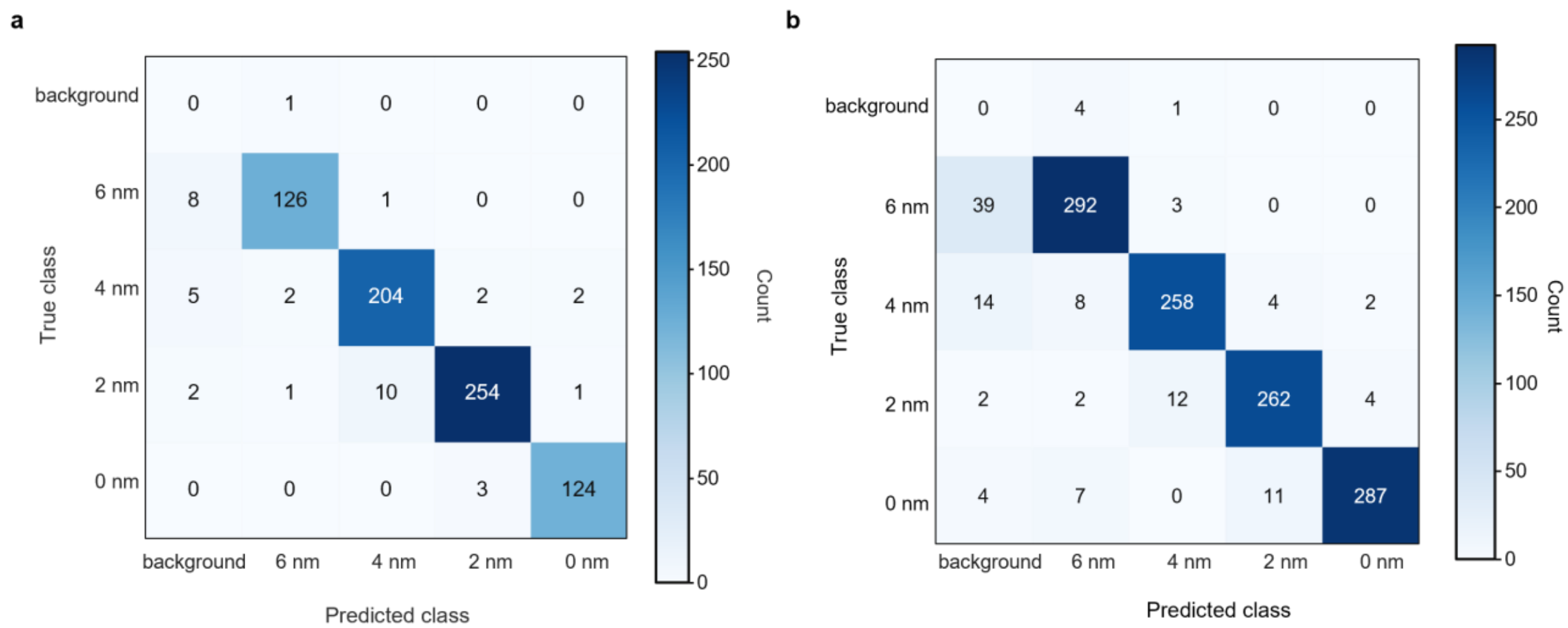

Figure S18 Pooled confusion-matrix analysis for apparent Z-height classification of Pt atoms on different supports. a, Pooled confusion matrix for apparent Z-height classification of Pt single atoms on the am-C support with local thickness variation. b, Pooled confusion matrix for apparent Z-height classification of Pt single atoms on the slightly tilted (~2°) crystalline BP support.

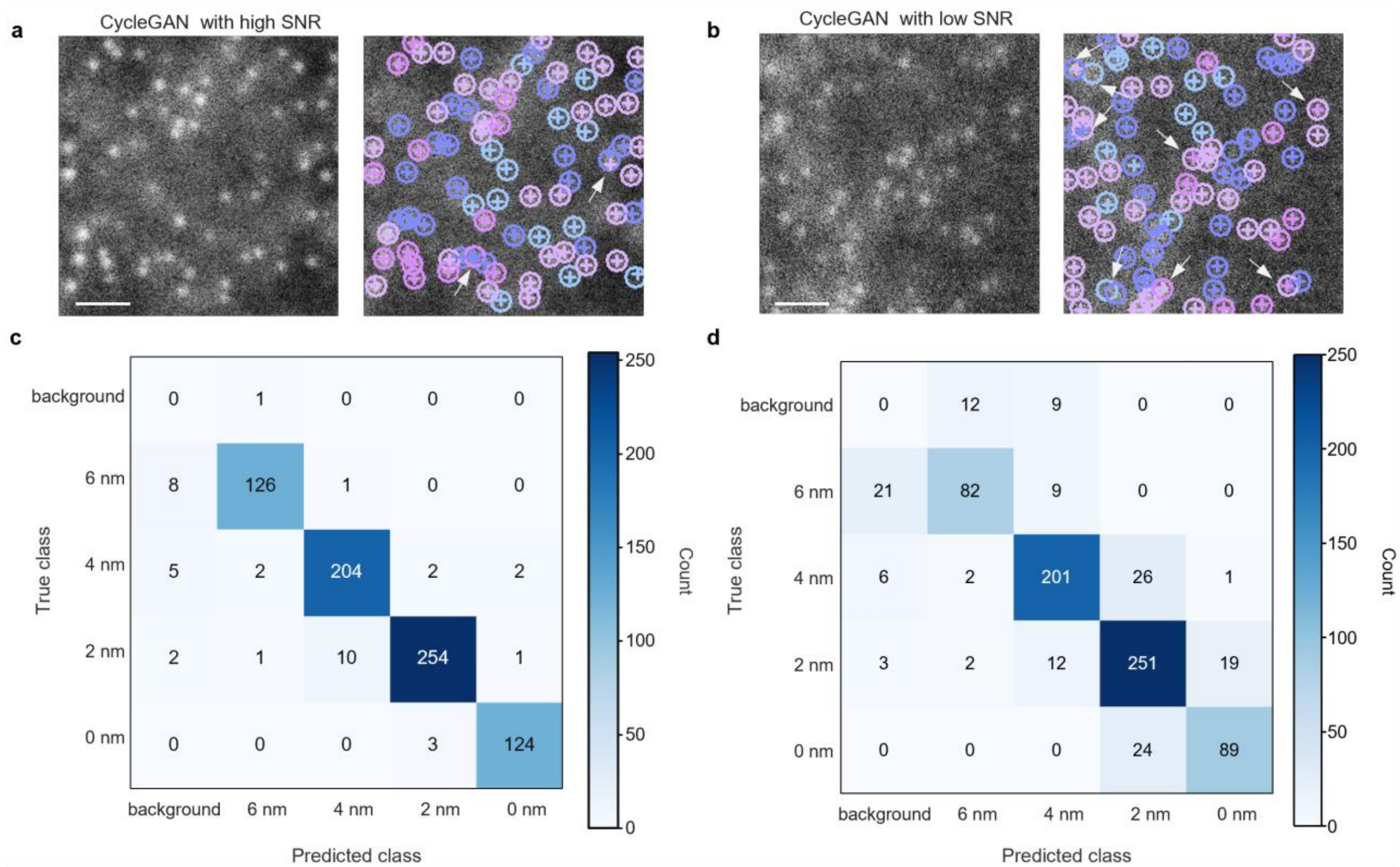

Figure S19 SNR-dependent pooled confusion-matrix analysis for apparent Z-height classification of simulated Pt atoms on am-C with thickness variation. a,b, Representative CycleGAN-processed simulated images (left) and corresponding GAN–UNet prediction overlays for Pt atoms (right) under the relatively high-SNR condition (a) and the low-SNR contaminated condition (b). Crosses and circles denote the ground truth and model predictions, respectively. c,d, Pooled confusion matrices for apparent Z-height classification of Pt atoms under relatively high-SNR conditions (c, SNR = −1.23 dB) and the low-SNR contaminated condition (d, SNR = −4.44 dB). The SNR values are reported in dB; a less negative value corresponds to higher image quality. The matrices were obtained by summing atom-level confusion matrices from all test images. Images in panel (a) and the pooled confusion matrix in panel (c) are intentionally reproduced from Figs. S16 and S18, respectively, as relatively high-SNR references for direct comparison with the low-SNR contaminated condition. Scale bars: 0.5 nm.

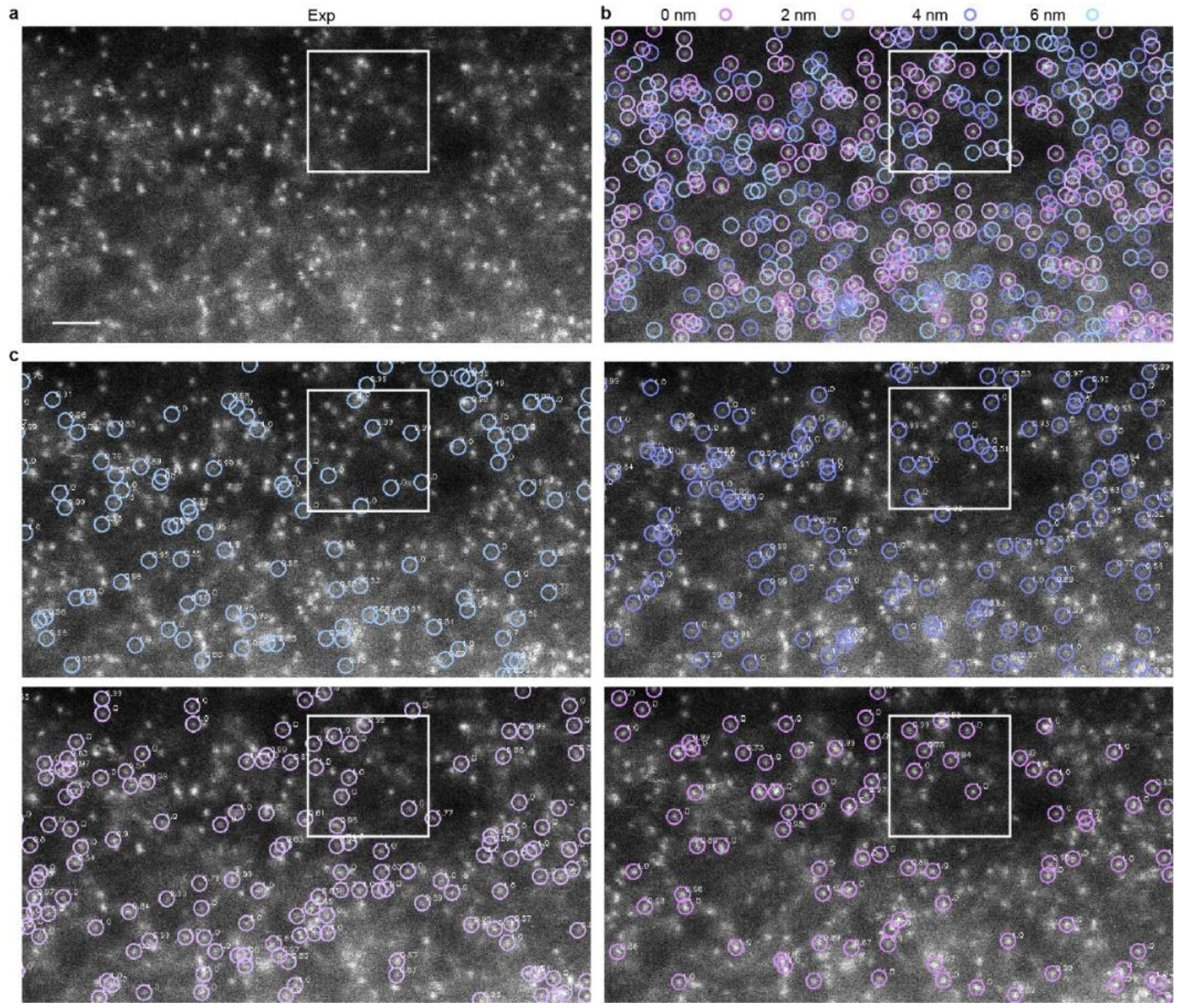

Figure S20 Automated height detection and identification of Pt atoms in experimental STEM images using GAN-U-Net-based models. a, Raw experimental STEM image. b, Atom height identification results, with detected atoms denoted by colored circles. c, Separated color maps representing different atom heights. The area outlined by the white frame represents the raw data in Fig. 4C. Scale bar, 1 nm.

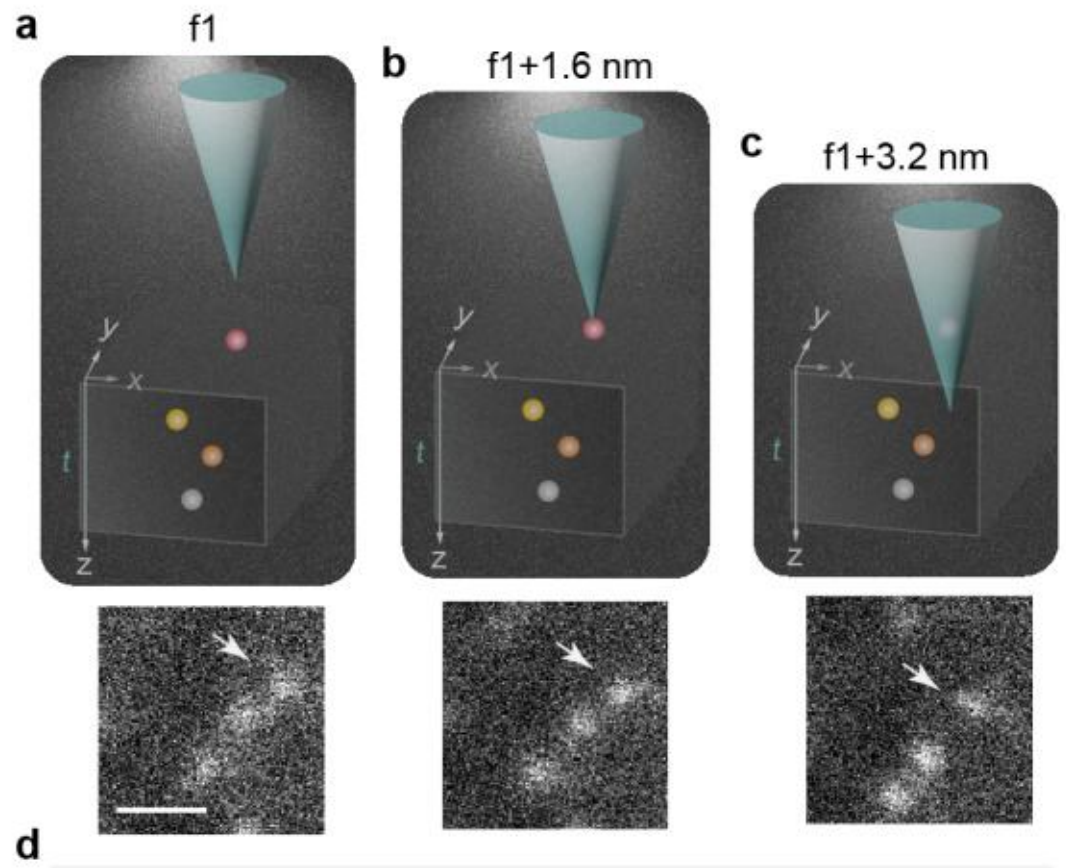

d

Defocus (nm)	f1	f1+1.6	f1+3.2
Intensity	134	143	124
Calibrated defocus (nm)	-1.6	0	1.6
Top surface		**Z = 0**	

Figure S21 Calibration of the experimental Z = 0 reference plane using fast through-focal ADF-STEM imaging. a–c, Schematic focal-plane positions and corresponding experimental ADF-STEM images of the same near-surface Pt single atom acquired at nominal defocus settings of f1 (a), f1 + 1.6 nm (b), and f1 + 3.2 nm (c). d, Summary of the nominal defocus settings, measured peak intensities, and calibrated defocus values. The f1 + 1.6 nm condition gives the highest Pt intensity and the sharpest apparent atomic profile, and was therefore assigned as the top-surface focal condition, defined as the experimental Z = 0 reference plane. Scale bars, 0.5 nm.

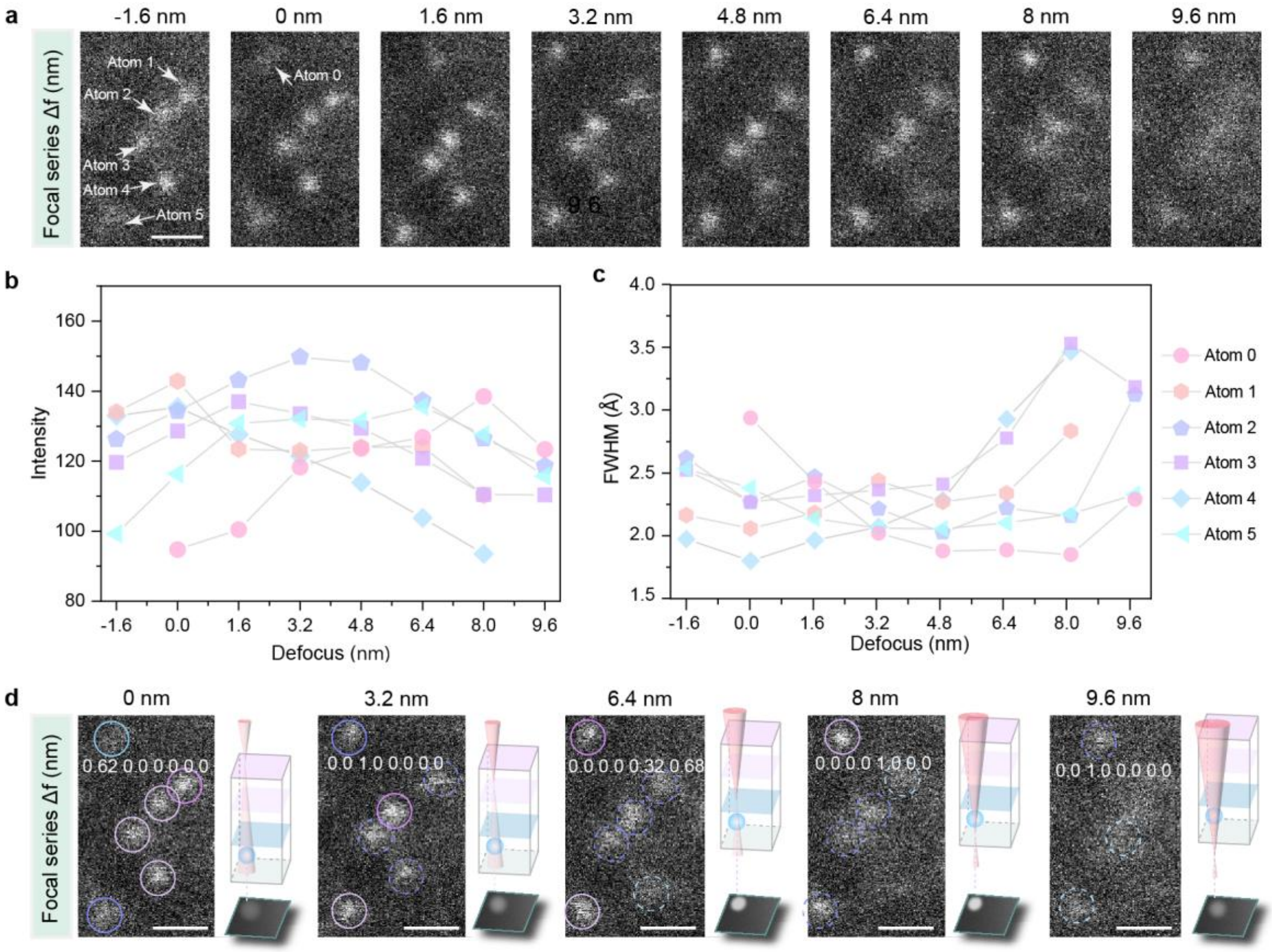

Figure S22 Through-focal validation of the experimentally calibrated Z = 0 reference plane. a, Representative through-focal ADF-STEM image series acquired consecutively from −1.6 to 9.6 nm relative to the calibrated Z = 0 reference. b, Peak-intensity evolution of representative Pt atoms across the focal series. c, FWHM evolution of the same representative Pt atoms across the focal series. d, ADF-STEM images at selected focal planes with model-predicted apparent Z-height labels overlaid. Solid circles indicate Pt atoms located below the focal plane, while dashed circles indicate Pt atoms located above the focal plane. Panel d is intentionally reproduced from Fig. 4E to link the main-text through-focal validation with the detailed quantitative analysis shown here. Scale bars: 0.5 nm.

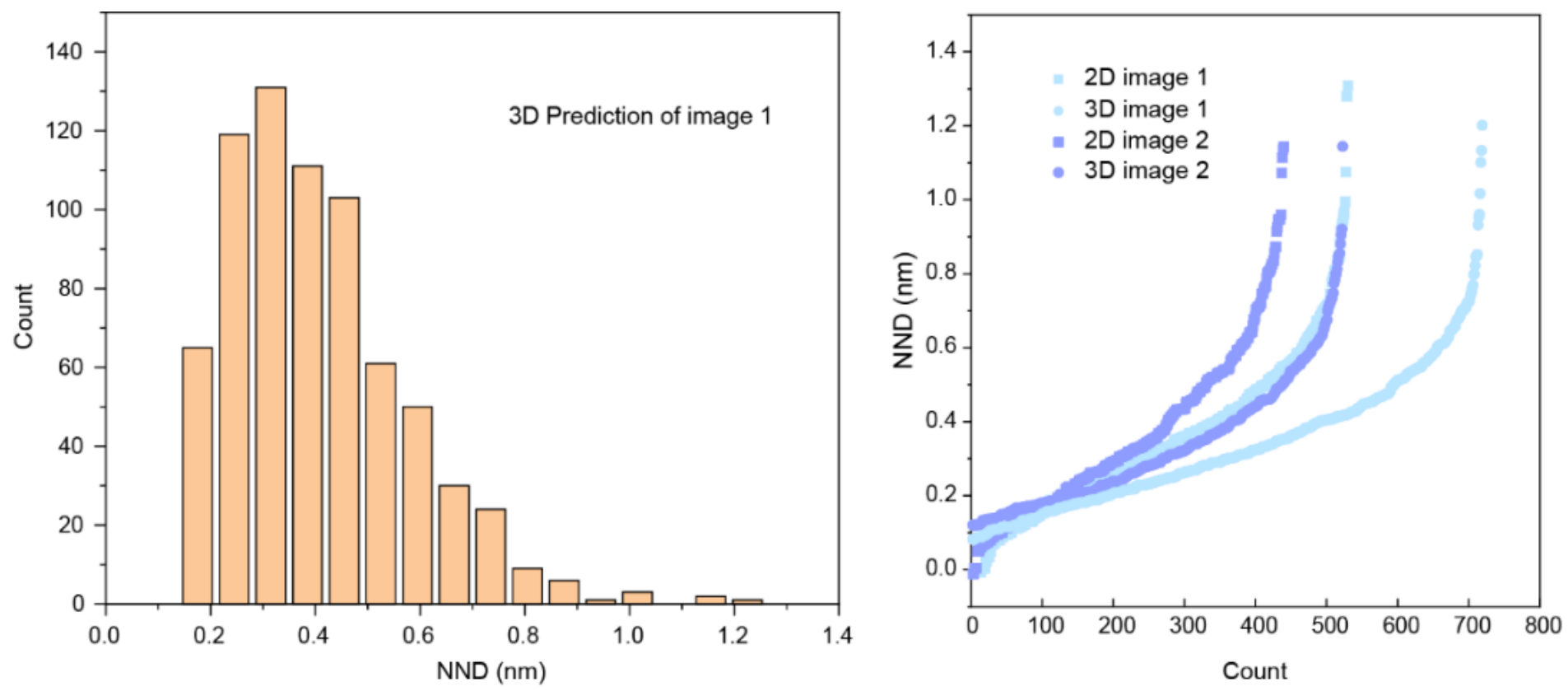

Figure S23 Nearest neighbor distance (NND) distribution of three-dimensional (3D) detected atoms on the sample from Fig. 3C (image 1) and Fig. S20 (image 2).

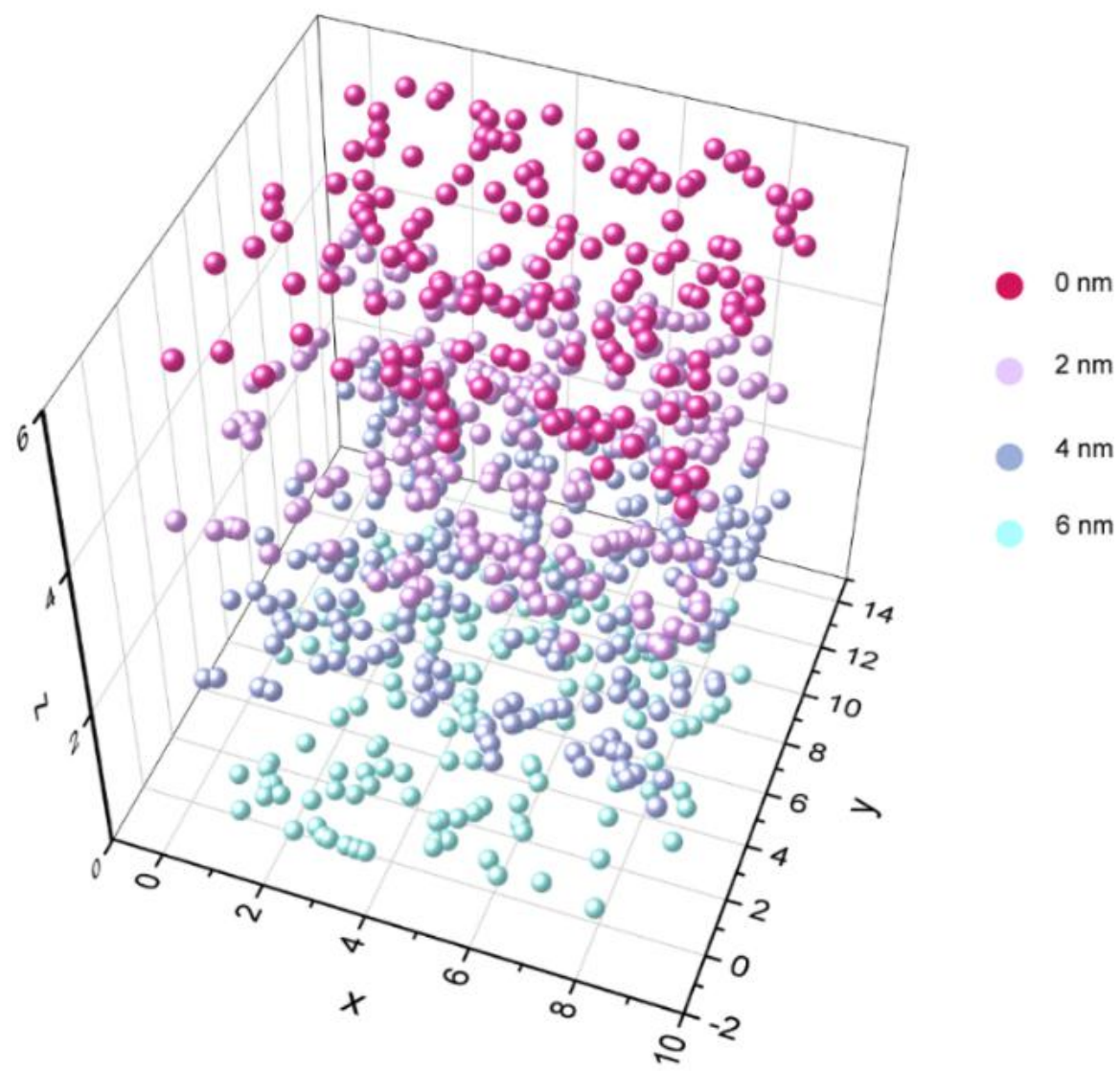

Figure S24 Reconstructed Pt atomic models in experimental STEM images from Fig. 3C using GAN-U-Net-based models.

Quinoline (2)

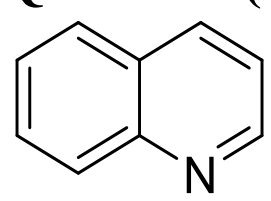

Yellow oil, 84% yield, 10.9 mg. ^{1}H NMR (400 MHz, $CDCl_3$) δ 8.86 (dd, J = 4.2, 1.8 Hz, 1H), 8.11 (d, J = 8.3 Hz, 1H), 8.06 (d, J = 8.5 Hz, 1H), 7.77 (d, J = 6.7 Hz, 1H), 7.66 (ddd, J = 8.4, 6.9, 1.5 Hz, 1H), 7.49 (ddd, J = 8.0, 6.8, 1.2 Hz, 1H), 7.35 (dd, J = 8.3, 4.2 Hz, 1H) ppm.

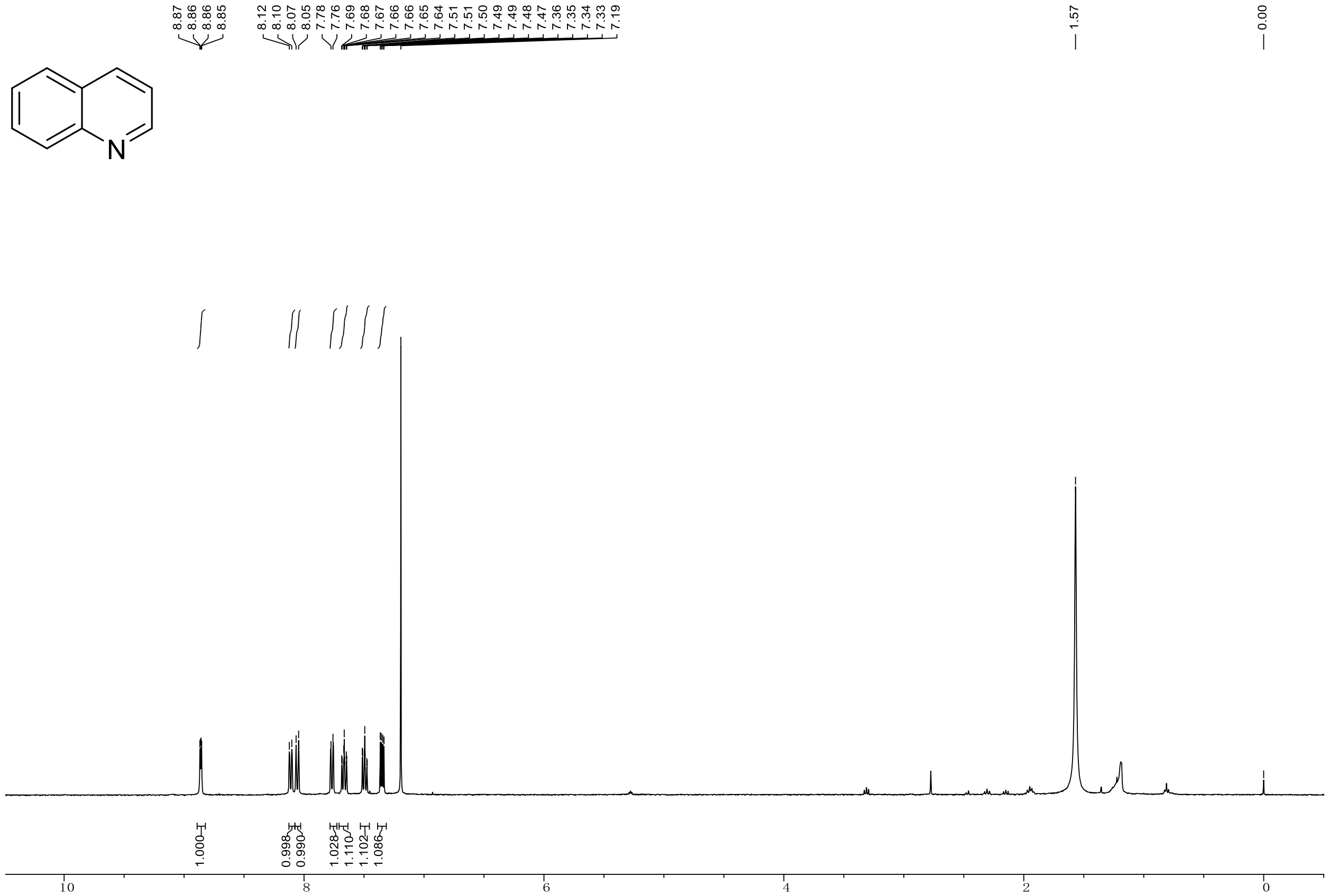

Figure S25 ^{1}H NMR spectrum (400 MHz, $CDCl_3$) of 2.

Isoquinoline (3)

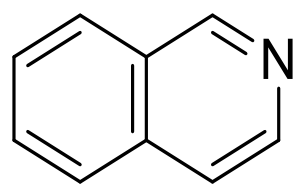

Yellow oil, 79% yield, 10.2 mg. ^{1}H NMR (400 MHz, $CDCl_3$) δ 9.20 (s, 1H), 8.46 (d, *J* = 5.8 Hz, 1H), 7.92 (d, *J* = 9.3 Hz, 1H), 7.77 (d, *J* = 7.2 Hz, 1H), 7.64 (ddd, *J* = 8.2, 6.9, 1.3 Hz, 1H), 7.60 (d, *J* = 5.8 Hz, 1H), 7.55 (ddd, *J* = 8.2, 6.8, 1.2 Hz, 1H) ppm.

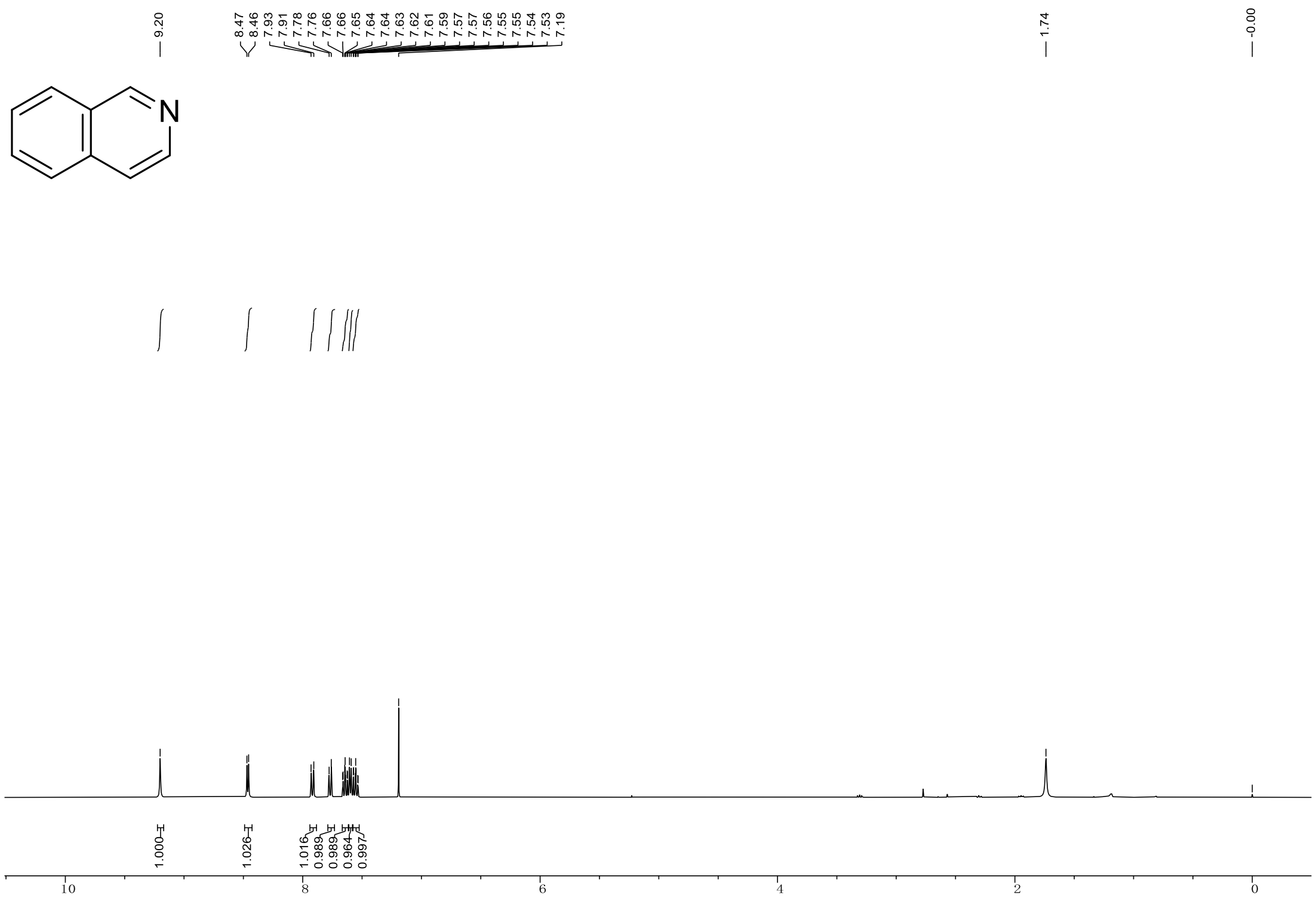

Figure S26 ^{1}H NMR spectrum (400 MHz, $CDCl_3$) of 3.

1*H*-indole (4)

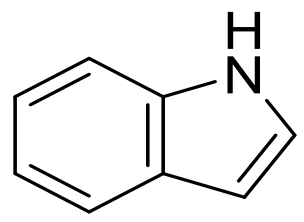

Yellow oil, 76% yield, 8.9 mg. ^{1}H NMR (400 MHz, $CDCl_3$) δ 8.08 (s, 1H), 7.58 (d, *J* = 7.9 Hz, 1H), 7.33 (d, *J* = 8.2 Hz, 1H), 7.15 – 7.10 (m, 2H), 7.05 (ddd, *J* = 8.0, 6.9, 1.1 Hz, 1H), 6.53 – 6.44 (m, 1H) ppm.

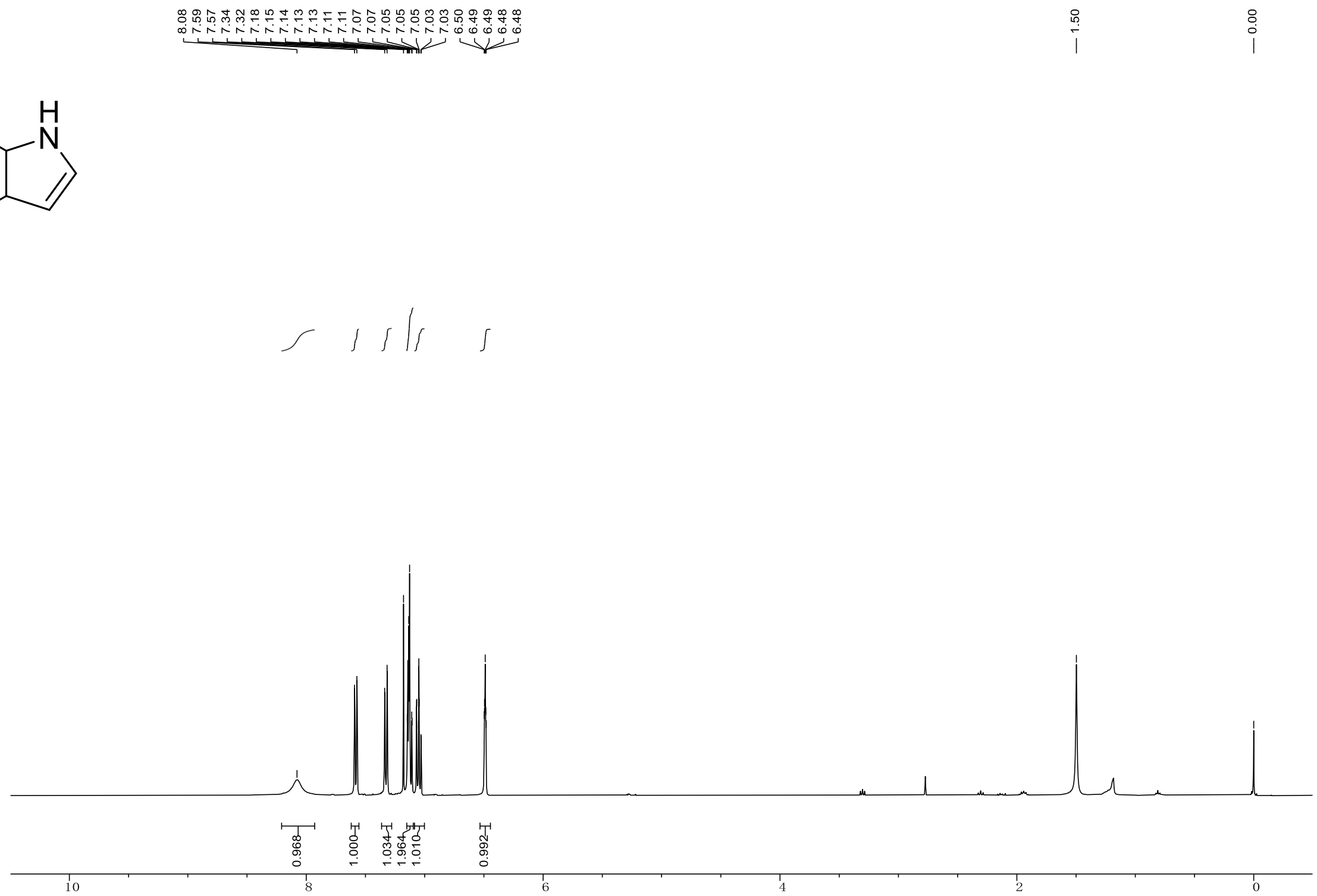

Figure S27 ^{1}H NMR spectrum (400 MHz, $CDCl_3$) of 4.

Table S1. Quantitative evaluation of the detection algorithm for the experimental Pt datasets, including precision, recall, and F1 scores, based on different training datasets

Data	Precision	Recall	F1
Sim	0.21	0.99	0.35
Manu	0.55	0.92	0.69
Gauss	0.43	0.92	0.58
UNIT	0.54	0.90	0.68
GAN	0.95	0.95	0.95

Table S2. Height-classification performance for apparent Z-height classification of simulated Pt atoms on am-C support with thickness variation

Depth	Average Precision	Average Recall	Average F1 Score
0 nm	0.98	0.98	0.98
2 nm	0.98	0.95	0.96
4 nm	0.95	0.95	0.95
6 nm	0.97	0.93	0.95
2D	1.00	0.98	0.99

Table S3. Height-classification performance for apparent Z-height classification of simulated Pt atoms on a slightly tilted (~2°) crystalline BP support

Depth	Average Precision	Average Recall	Average F1 Score
0 nm	0.98	0.93	0.95
2 nm	0.95	0.93	0.94
4 nm	0.94	0.90	0.92
6 nm	0.93	0.87	0.90
2D	1.00	0.95	0.97

Table S4. Row-normalized confusion matrix for apparent Z-height classification of simulated Pt atoms on the am-C support with thickness variation

True \ Predicted	0 nm	2 nm	4 nm	6 nm	background
0 nm	**97.64%**	**2.36%**	0.00%	0.00%	0.00%
2 nm	0.37%	**94.78%**	**3.73%**	0.37%	0.75%
4 nm	0.93%	0.93%	**94.88%**	0.93%	**2.33%**
6 nm	0.00%	0.00%	0.74%	**93.33%**	**5.93%**

Table S5. Row-normalized confusion matrix for apparent Z-height classification of simulated Pt atoms on a slightly tilted (~2°) crystalline BP support

True \ Predicted	0 nm	2 nm	4 nm	6 nm	background
0 nm	**92.88%**	**3.56%**	0.00%	2.27%	1.29%
2 nm	1.42%	**92.91%**	**4.26%**	0.71%	0.71%
4 nm	0.70%	1.40%	**90.21%**	2.80%	**4.90%**
6 nm	0.00%	0.00%	0.90%	**87.43%**	**11.68%**

Table S6. Row-normalized confusion matrix for apparent Z-height classification of simulated Pt atoms on am-C support with local thickness variation under the low-SNR contaminated condition

True \ Predicted	0 nm	2 nm	4 nm	6 nm	background
0 nm	**78.76%**	**21.24%**	0.00%	0.00%	0.00%
2 nm	**6.62%**	**87.46%**	4.18%	0.70%	1.05%
4 nm	0.42%	**11.02%**	**85.17%**	0.85%	2.54%
6 nm	0.00%	0.00%	8.04%	**73.21%**	**18.75%**

SNR was calculated using a reference-based image-power definition, as described in Eqs. (6)–(9). In this calculation, the reference image $r$represents the ideal noise-free ground-truth image, whereas the test image $t$represents the noisy or evaluated image. The signal power was defined as the mean-squared intensity of the reference image, and the noise power was defined as the mean-squared difference between the test and reference images. The SNR was then expressed in decibels as follows:

$$P_{\text{signal}} = \frac{1}{N} \sum_{i=1}^{N} {r_i}^2 \quad (6)$$

$$P_{\text{noise}} = \frac{1}{N} \sum_{i=1}^{N} (t_i - r_i)^2 \quad (7)$$

$$\text{SNR (dB)} = 10 \log_{10} \left(\frac{P_{\text{signal}}}{P_{\text{noise}}}\right) \quad (8)$$

Equivalently, because the same number of pixels is used in both mean operations, this expression can also be written as:

$$\text{SNR (dB)} = 10 \log_{10} \left[\frac{\sum_{i=1}^{N} {r_i}^2}{\sum_{i=1}^{N} (t_i - r_i)^2}\right] \quad (9)$$

Here, N is the total number of pixels, and r_i and t_i are the pixel intensities of the reference and test images, respectively. When $P_{\text{noise}} = 0$, indicating that the test image is identical to the reference image, the SNR is treated as $+\infty$.

Table S7. Height-classification performance of simulated Pt atoms on the uniform am-C support with a depth step of 1 nm

Depth	Average Precision	Average Recall	Average F1 Score
0 nm	0.91	0.95	0.93
1 nm	0.82	0.84	0.83
2 nm	0.90	0.81	0.85
3 nm	0.87	0.85	0.86
4 nm	0.87	0.75	0.80
5 nm	0.81	0.87	0.84
6 nm	0.89	0.86	0.88
2D	1.00	0.97	0.98
